# Synthesis and Antibacterial Activity of Novel Triazolo[4,3-*a*]pyrazine Derivatives

**DOI:** 10.3390/molecules28237876

**Published:** 2023-11-30

**Authors:** Zhang Hu, Hongrui Dong, Zhenyu Si, Yurong Zhao, Yuanwei Liang

**Affiliations:** Faculty of Chemistry and Environmental Science, Guangdong Ocean University, Zhanjiang 524088, China; huzhang@gdou.edu.cn (Z.H.);

**Keywords:** nitrogen-containing heterocycles, triazole compounds, triazolo[4,3-*a*]pyrazine derivatives, antibacterial activity

## Abstract

Infectious diseases pose a major challenge to human health, and there is an urgent need to develop new antimicrobial agents with excellent antibacterial activity. A series of novel triazolo[4,3-*a*]pyrazine derivatives were synthesized and their structures were characterized using various techniques, such as melting point, ^1^H and ^13^C nuclear magnetic resonance spectroscopy, mass spectrometry, and elemental analysis. All the synthesized compounds were evaluated for in vitro antibacterial activity using the microbroth dilution method. Among all the tested compounds, some showed moderate to good antibacterial activities against both Gram-positive *Staphylococcus aureus* and Gram-negative *Escherichia coli* strains. In particular, compound **2e** exhibited superior antibacterial activities (MICs: 32 μg/mL against *Staphylococcus aureus* and 16 μg/mL against *Escherichia coli*), which was comparable to the first-line antibacterial agent ampicillin. In addition, the structure–activity relationship of the triazolo[4,3-*a*]pyrazine derivatives was preliminarily investigated.

## 1. Introduction

In recent years, infectious diseases have posed major challenges to human health. This widespread infection reveals a gradual decrease of sensitivity to the currently used antimicrobial agents [1]. Moreover, the antimicrobial agents on the market have various drawbacks, such as narrow antimicrobial spectrum, limited activity, potential toxicity, and gradually developing microbial resistance [2]. Therefore, developing new compounds with excellent antibacterial activity has always been one of the most challenging tasks in the antibacterial field.

Nitrogen-containing heterocycles are widely found in natural products, synthetic drugs, and functional materials, and are the basic backbone of many physiologically active compounds and drugs. In the pharmaceutical field, in addition to biologically active natural products, some synthetic nitrogen-containing heterocyclic compounds have become popular drugs, such as chlorpromazine for psychosis, captopril for hypertension, delorazepam for anxiety, and isoniazid for tuberculosis. It is estimated that more than 80% of the best-selling small-molecule drugs contain at least one nitrogen heterocycle [3,4].

Nitrogen-containing heterocyclic chemistry is the most challenging and fruitful area of anti-infective drugs, among which triazoles are one of the most important heterocyclic compounds due to their excellent pharmacological activities as essential components of all cells and organisms [5,6]. The synthesis and drug screening of triazole compounds have been of great interest. This is because the unique chemical structure of the triazole moiety can affect the polarity, lipophilicity, and hydrogen-bond-forming ability of compounds to improve their pharmacokinetics, pharmacology, and toxicology. Therefore, triazole compounds have high activity and low toxicity, as well as good pharmacokinetic and pharmacodynamic characteristics [7]. Various antimicrobial triazole derivatives have been developed and most of them show potent antimicrobial activity [8]. It is particularly noteworthy that more effective antibacterial candidates might be obtained from the various triazole fused heterocyclic derivatives, extensively existing in chemistry, such as triazolo[1,5-*a*]pyrimidine [9], triazolo[4,5-*c*]pyridine [10], triazolo[4,3-*a*]pyrazine [11], triazolo[4,5-*d*]pyrimidine [12], triazolo[4,3-*a*]quinoxaline [13], triazolo[1,5-*a*]quinolines [14], and triazolo[1,5-*a*]pyridine [15,16]. Some triazole fused heterocyclic derivatives have been found to have high antibacterial activity against both drug-sensitive and drug-resistant pathogens and are not inferior to the first-line antimicrobial agents [17,18].

**Figure 1:**
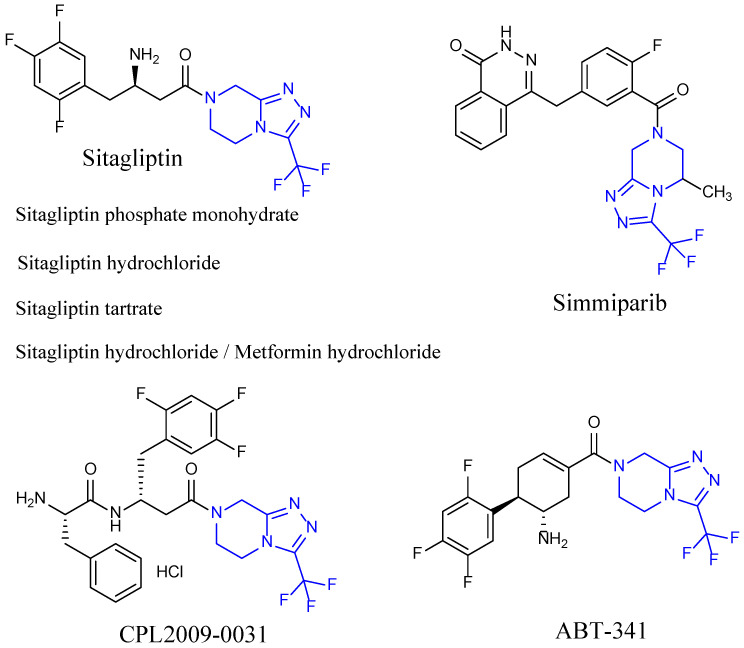
Seven drugs bearing trifluoromethyl triazolo[4,3-*a*]pyrazine moiety (in blue) on the market.

Triazolo[4,3-*a*]pyrazine is a privileged building block/scaffold used for the synthesis of biologically active molecules and has received significant attention in the field of synthetic organic chemistry. Triazolo[4,3-*a*]pyrazine derivatives have a wide range of biological activities, such as antidiabetic [19], anti-platelet aggregations [20], anti-fungal [21], anti-bacterial [22], anti-malarial [23], anti-tubercular [24], and anticonvulsant properties [25], which are important in drug discovery programs. 3-(Trifluoromethyl)-1,2,4-triazolo[4,3-*a*]pyrazine is the key pharmacophore of sitagliptin phosphate, a new drug for the treatment of type II-diabetes mellitus. According to the Pharmacodia Global Database (https://data.pharmacodia.com/, accessed on 15 October 2023), based on the 3-(trifluoromethyl)-1,2,4-triazolo[4,3-*a*]pyrazine moiety, there are seven marketed drugs (Figure 1), including four Sitagliptin drugs (Sitagliptin phosphate monohydrate, Sitagliptin hydrochloride, Sitagliptin tartrate, and Metformin hydrochloride/Sitagliptin hydrochloride), Simmiparib, CPL2009-0031, and ABT-341; there are also 302 reported active molecules, including 182 small molecules (with a molecular weight less than 500 Da). 

As a continuation of our studies to search for bioactive molecules and synthetic heterocyclic compounds, herein, some novel triazolo[4,3-*a*]pyrazine derivatives were synthesized via two different routes using trifluoromethyl 1,2,4-triazolo[4,3-*a*]pyrazine as the main scaffold, and their antibacterial activities were evaluated.

## 2. Results and Discussion

### 2.1. Chemistry

**Scheme 1:**
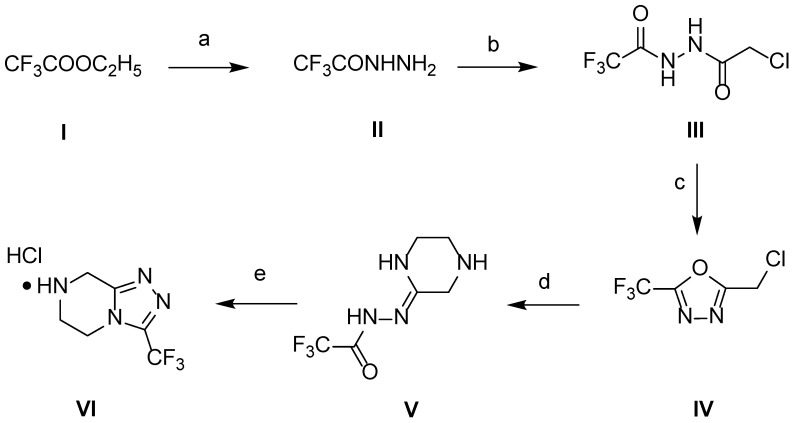
Synthesis of 3-(trifluoromethyl)-1,2,4-triazolo[4,3-*a*]pyrazine as the key scaffold. Reagents and conditions: (a) 35% NH_2_NH_2_·H_2_O, CH_3_CN, 20 °C, 1 h; (b) NaOH (50%), ClCH_2_COCl, CH_3_CN, 10 °C, 3 h; (c) POCl_3_, CH_3_CN, 80 °C, 24 h; (d) 3 equiv. of H_2_NCH_2_CH_2_NH_2_, MeOH, −20 °C, 1 h; (e) Con. HCl, MeOH, 55 °C, 1 h.

The synthetic route of 3-(trifluoromethyl)-1,2,4-triazolo[4,3-*a*]pyrazine as a key scaffold is presented in Scheme 1 [21,26]. The starting material, ethyl trifluoroacetate (**I**), was dissolved in acetonitrile and reacted with hydrazine hydrate (35%, *w*/*v*) for 1 h under almost room-temperature (20 °C) conditions to obtain trifluoroacetohydrazide (**II**). Without separation treatment, sodium hydroxide solution (50%, *w*/*v*) and chloroacetyl chloride solution were individually added dropwise to the solution of **II** using constant-pressure dropping funnels. The reaction was carried out at a low temperature (10 °C) for 3 h, and the intermediate **III** precipitated. In the presence of phosphorus oxide trichloride, intermediate **III** was dehydrated via heating and a cyclization reaction occurred to yield the intermediate oxadiazole **IV**. Under low-temperature conditions (−20 °C), ethylenediamine attacked as a nucleophile, and oxadiazole **IV** was first ring-opened and subsequently cyclized to obtain compound **V**. Finally, the intermediate **V** further cyclized in the presence of concentrated hydrochloric acid to obtain the key scaffold 3-(trifluoromethyl)-1,2,4-triazolo[4,3-*a*]pyrazine (**VI**) in good yield. The structure of **VI** was confirmed using melting point, ^1^H nuclear magnetic resonance (^1^H NMR) spectroscopy, and mass spectrometry (MS), all of which were in agreement with previous reports [21,26].

**Scheme 2:**
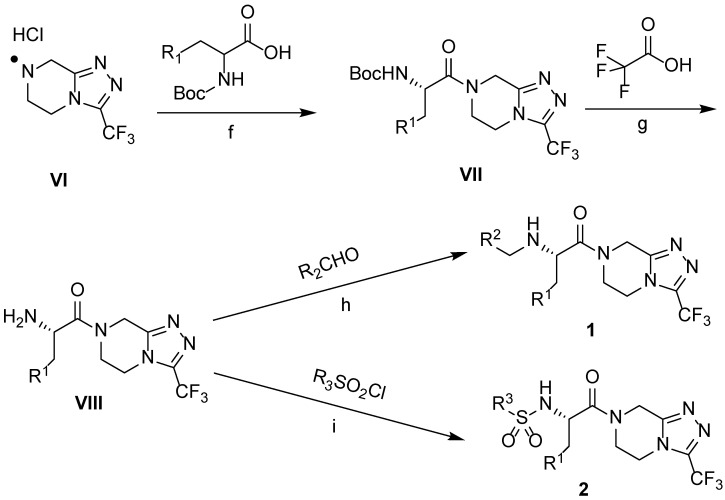
Synthesis of triazolo[4,3-*a*]pyrazine derivatives. Reagents and conditions: (f) DCC, Et_3_N, DCM, 0 °C to r.t, 36 h; (g) DCM, r.t, 4 h; (h) NaBH_3_CN, CH_3_COOH, MeOH, 0 °C to r.t, overnight; (i) Et_3_N, DCM, 0 °C to r.t, 3 h.

The synthetic routes of target triazolo[4,3-*a*]pyrazine derivatives **1** and **2** are shown in Scheme 2. Using dichloromethane (DCM) as a solvent, and in the presence of triethylamine, t-butyloxycarbonyl (Boc)-protected amino acids underwent an amide condensation reaction with scaffold **VI** through the activation of DCC to obtain intermediate **VII**. Subsequently, the reaction was carried out in a mixed solution of trifluoroacetic acid and DCM (1:1, *v*/*v*) at room temperature for 4 h, and amino deprotection occurred to yield intermediate **VIII**. Finally, two types of products were obtained from intermediate **VIII** via two reaction approaches. One involved the nucleophilic addition of intermediate **VIII** to an aldehyde in methanol solvent in the presence of acetic acid, followed by the removal of one water molecule to yield the imine intermediate, which was reduced under the action of NaBH_3_CN to obtain the target product **1**. The other was the sulfonylation of intermediate **VIII** with sulfonyl chloride in DCM solvent in the presence of triethylamine to give the desired product **2** in good yield. The products, with various chemical structures, are listed in Table 1; they were characterized using various techniques, such as melting point, ^1^H and ^13^C NMR spectroscopy, MS, and elemental analysis (Figures S1–S23).

The first substituted site (R^1^) in the structure of the target product was mainly derived from changes in amino acids. For ease of synthesis, Boc-protected L-phenylalanine and L-tryptophan, two natural amino acids with strong absorption in the ultraviolet region, were chosen, which allowed us to monitor the reaction progress with thin layer chromatography. For the second substituted site (R^2^), various types of aldehydes were selected as the reaction reagents, including aliphatic aldehydes, aromatic aldehydes, and heterocyclic aromatic aldehydes. For the third site (R^3^), the selected reagents were aliphatic sulfonyl chloride, aromatic sulfonyl chloride, and heterocyclic sulfonyl chloride. In the reduction amination reaction of step h, the molar ratio of amine to aldehyde had a relatively large influence on the purity of the target product. When the molar ratio of amine to aldehyde was 1:1, a small amount of *N*,*N*-disubstituted by-products formed; when the molar ratio of amine to aldehyde was 1.2:1, the target product obtained was of higher purity. In the sulfonylation reaction of step i, when the R^1^ group was a 3-indolyl group, the selectivity of the reaction needed to be controlled because acylable sites also existed in indole residues. Due to the relatively weak reactivity of indole residues, the sulfonylation selectivity could be controlled by slowly adding the sulfonylation reagent under low-temperature conditions (0 °C). Meanwhile, to facilitate purification of the product, a slight excess of reactant **VIII** can be used (molar ratio of **VIII** to sulfonylation reagent = 1.1:1), which avoided the generation of by-products from sulfonylation of indole residues as much as possible.

### 2.2. Antibacterial Activity

The antibacterial activities of fifteen newly synthesized triazolo[4,3-*a*]pyrazine derivatives were tested using the microbroth dilution method, and their minimum inhibitory concentrations (MICs) against *Staphylococcus aureus* (*S. aureus*) and *Escherichia coli* (*E. coli*) strains were obtained. The results are shown in Table 2; a smaller MIC means a better antibacterial effect of the compound. The MIC values of ampicillin as a positive control against *S. aureus* and *E. coli* were 32 and 8 μg/mL, respectively, which were similar to previously reported results (MICs: 35 μg/mL against *S. aureus* and 10 μg/mL against *E. coli*) [27,28]. Against *S. aureus*, most of the tested compounds exhibited slight or good antibacterial activity (32–256 μg/mL), among which the MIC values of compounds **1a**, **1f**, **1i**, **2c**, and **2e** (32–64 μg/mL) were the same or very close to that of the positive control. Meanwhile, most compounds showed slight antibacterial activity against *E. coli* (MIC = 64–256 μg/mL), whereas the MIC values (16–32 μg/mL) of compounds **1f**, **1i**, and **2e** were close to that of the positive control. Compound **2e** showed the strongest antibacterial activity against *E. coli* (MIC = 16 μg/mL), which was comparable to the positive control ampicillin. 

**Figure 2:**
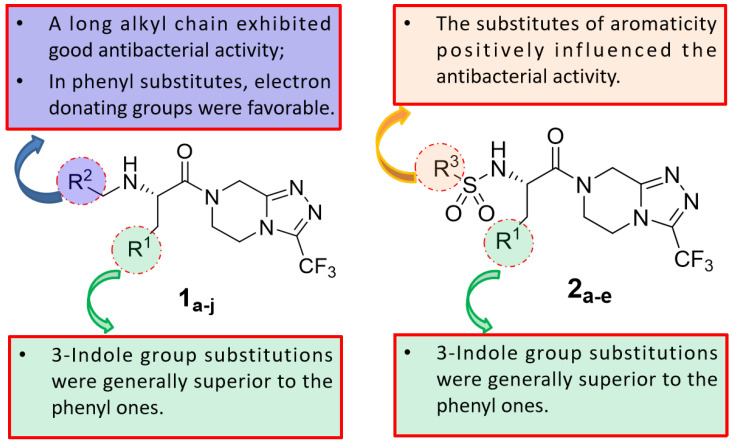
The preliminary analysis on the structure–activity relationship of triazole[4,3-*a*]pyrazine derivatives.

The structure–activity relationship of triazolo[4,3-*a*]pyrazine derivatives was preliminarily analyzed and the results were summarized in Figure 2. In general, [1,2,4]triazolo[4,3-*a*]pyrazine nucleus bearing a ethylenediamine moiety were prone to the antibacterial activities. It may be because that at physiological pH conditions, protonated amines or nitrogen heterocycles could form π-cation interactions with the amino acid carbonyl groups of DNA gyrase [29], which was beneficial for antibacterial effects. Compounds with R^1^ as a 3-indole group were generally more active than phenyl-substituted ones. Liu et al. demonstrated that indole derivatives exhibit moderate to good antibacterial effects, but *N*-methylation of the indole moiety was not necessary for a compound to have good antibacterial activity [30]. From these results, it could be reasonably inferred that triazolo[4,3-*a*]pyrazine derivatives with an indole moiety might form hydrogen bond interactions with key amino acid residues of the target receptor to enhance antibacterial effects. At the R^2^ site, for the antibacterial activity of compounds, long alkyl chain groups were superior to the aromatic groups, such as compounds **1a** and **1f**, and the similar conclusion was also drawn by Kaur et al. [31] and Pokhodylo et al. [32]. Among phenyl-substituted compounds at the R^2^ site, bearing electron-donating groups favored the antibacterial activity as expressed in the compounds **1d** and **1i** [33]. When R^2^ substituent group is an aliphatic chain, it can increase the lipophilic nature of the compounds, thereby making the molecules more cell permeable [34]. When R^2^ is an aromatic one, such as phenyl, it can produce π-π stacking with the active site [35]. For the R^3^ site, the aromaticity of substituted groups positively influenced the antibacterial activity as expressed in the compounds **2b**, **2c** and **2e**, which was in agreement with previous reports [36].

Cell membrane of bacteria is a natural selective permeation barrier that prevents harmful substances, such as toxins, drugs, and damaging enzymes, from entering and allows nutrients to penetrate cells [37]. Chen et al. synthesized some novel 1,2,4-triazole Schiff base derivatives that demonstrated a good inhibitory effect against the tested bacteria. The study of sterilization process demonstrated the possibility of destroying the bacterial cell membrane structure, and thus achieving inhibition [38]. DNA gyrase is one of the key enzymes among the entire bacterial species, and its inhibition will lead to interruption of DNA synthesis, followed by cell death. Therefore, DNA gyrase inhibition is one of the most effective approaches in antibacterial chemotherapy [39,40]. Pathak et al. [41] developed eighteen novel thiazolidine-1,2,4-triazole derivatives with significant-to-moderate antibacterial activity. A molecular docking study indicated that the synthesized derivatives could bind to DNA gyrase to inhibit its function. Topoisomerase IV keeps DNA in a proper topological state during DNA replication and transcription; it is a clinically validated, highly effective drug target [42]. A series of 1,2,4-triazole-norfloxacin hybrid compounds were synthesized and their in vitro antibacterial activity against common pathogens was evaluated, most of which exhibited antibacterial activity superior to that of norfloxacin. Molecular docking showed that the compounds had remarkable affinity for the bacterial topoisomerase IV [43]. In a word, 1,2,4-triazole derivatives could exert their antibacterial effects through destroying the bacterial cell membrane structure, and inhibiting DNA gyrase and topoisomerase IV, and play an important role in the discovery of novel antibacterial agents. The newly synthesized fifteen triazolo[4,3-*a*]pyrazine derivatives showed different antibacterial activity against *S. aureus* and *E. coli*. Among them, compound **2e** displayed superior antibacterial activity (MICs: 32 μg/mL against *S. aureus* and 16 μg/mL against *E. coli*), which was comparable to ampicillin. It might be due to its good capacity of binding with the target receptor so as to possess good antibacterial property, suggesting that compound **2e** was worth being further optimized.

## 3. Materials and Methods

### 3.1. Materials

Boc-protected L-phenylalanine and L-tryptophan, as well as various sulfonyl chlorides, were obtained on a commercial basis. All other chemicals and chemical reagents were purchased from Shanghai Aladdin Biochemical Technology Co., Ltd. (Shanghai, China), and used directly without further treatment unless otherwise stated. *E. coli* ATCC 8739 and *S. aureus* ATCC 6538 were gained from Guangdong Microbial Culture Collection Center, Guangzhou, China.

### 3.2. General Characterization

All the synthesized compounds were characterized. The melting points (mp) were measured on a melting point instrument (Büchi B-540) and were uncorrected. ^1^H NMR and ^13^C NMR spectra were recorded on a Bruker Avance III 500 MHz spectrometer with CDCl_3_ or DMSO-d6 as solvents. Chemical shifts and spin-spin coupling constants were provided in ppm and Hz, respectively. Mass spectra were recorded on a Finnigan Mat LCQ mass spectrometer with an electron spray ionization (ESI) technique. Elemental analyses were carried out on a PE-2400 CHN analyzer (PerkinElmer Inc., Waltham, MA, USA).

### 3.3. Synthesis of N′-(2-chloroacetyl)-2,2,2-trifluoroacetohydrazide (***III***)

Hydrazine hydrate (0.3 mol, 35%, *w*/*v*) and acetonitrile (210 mL) were added to a 500 mL three-necked flask and stirred in an ice water bath. Ethyl trifluoroacetate (0.303 mol, 43.05 g) was slowly added dropwise to the reaction flask, and the internal temperature was kept below 10 °C. After the dropwise addition, the reaction was carried out at 20 °C for 1 h. The reaction flask was then placed on crushed ice, 50% aqueous NaOH (0.305 mol, 12.22 g) solution and chloroacetyl chloride (0.305 mol, 34.45 g) were added dropwise to the flask simultaneously using constant-pressure dropping funnels, the internal temperature was kept below 10 °C, and the reaction was maintained for 3 h. The solvent was evaporated by rotary evaporation at 25 °C under reduced pressure till the volume was 1/3 of the original. The mixture was then filtered and washed twice with acetonitrile. Afterward, the solvent was evaporated under reduced pressure to obtain a pink solid, which was then recrystallized with chloroform to yield white solids. Yield 85%, mp 139–141 °C (lit [21] 139–140 °C). ^1^H NMR (DMSO-d6) δ: 11.69 (1H, s), 10.69 (1H, s), 4.22 (2H, s).

### 3.4. Synthesis of 2-(chloromethyl)-5-(trifluoromethyl)-1,3,4-oxadiazole (***IV***)

Intermediate **III** (43 g, 212 mmol) was added to a flask with 60 mL of anhydrous acetonitrile and freshly treated phosphorus oxide trichloride (32.5 g, 212 mmol). The mixture was heated to 80 °C and kept for 24 h before cooling to room temperature. Then, a preformulated and precooled quenching solution (300 mL of methyl tert-butyl ether (MTBE) mixed with 300 mL of water) was added to the reaction mixture and stirred for 0.5 h to separate the organic phase. The organic phase was then washed with 5% KHCO_3_ solution and 20% saline, then the solvent was rotary evaporated at 25 °C under reduced pressure to obtain an orange liquid, which was then distilled under reduced pressure to obtain a colorless transparent liquid. Yield 61%. ^1^H NMR (CDCl_3_) δ: 4.76 (2H, s).

### 3.5. Synthesis of 2,2,2-trifluoro-N′-(piperazin-2-ylidene)acetohydrazide (***V***)

Ethylenediamine (2.71 g, 45.0 mmol) was placed in a 50 mL flask, followed by adding methanol (15 mL). The mixture was set on an ice–salt bath with stirring, and the intermediate **IV** (2.8 g, 15.0 mmo) was added dropwise. After reacting at −20 °C for 1 h, the reactants turned from a clear solution to a slurry. Then, ethanol (25 mL) was added, and the mixture was stirred and subjected to suction filtration. The filter cake was washed with −5 °C ethanol, and vacuum dried to obtain a white solid. Yield 68%, mp 138–140 °C (lit [26] 141 °C). ^1^H NMR (DMSO-d6) δ: 3.63 (2H, s), 3.17 (2H, t), 2.87 (2H, t).

### 3.6. Synthesis of 3-(trifluoromethyl)-5,6,7,8-tetrahydro-[1,2,4]triazolo[4,3-a]pyrazine Hydrochloride Salt (***VI***)

Intermediate **V** (20.2 g, 96 mmol) was added to a flask containing methanol (80 mL) and stirred. The mixture presented as a white slurry and was heated to 55 °C. Then, 37% concentrated hydrochloric acid (97 mmol) was slowly added dropwise to the flask, and the mixture was kept for 1 h at 55 °C. The solvent was partially evaporated at 20 °C under reduced pressure until the crystals precipitated. The mixture was left overnight and filtered, and the filter cake was washed with precooled MTBE/ethanol (3:1) and dried in a vacuum to give a white solid. Yield 78%, mp 260–262 °C (lit [21] 263–265 °C). ^1^H NMR (DMSO-d6) δ: 10.34 (2H, s), 4.62 (2H, s), 4.43 (2H, t), 3.63 (2H, t). ESI-MS (*m*/*z*): calculated for C6H7F3N4, [M]^+^: 192.15; found 192.81.

### 3.7. Synthesis of Tert-Butyl (S)-(1-oxo-3-phenyl-1-(3-(trifluoromethyl)-5,6-dihydro-[1,2,4]triazolo[4,3-a]pyrazin-7(8H)-yl)propan-2-yl)carbamate (***VIIa***)

Boc-L-phenylalanine (0.5g, 1.9 mmol) and DCM (20 mL) were added to a 25 mL flask, stirred to dissolve, and placed on ice to cool to 0 °C. Then, DCC (0.433 g, 2.1 mmol) was added under a nitrogen atmosphere and the mixture was stirred for 1 h. Intermediate **VI** (0.6 g, 2.66 mmol) and triethylamine (296 mg, 2.93 mmol) were added to the flask, and the reaction proceeded at room temperature for 36 h before filtration. The organic phase was washed with water and brine, and dried over anhydrous Na_2_SO_4_. The organic phase was then filtered, and the filtrate was concentrated under reduced pressure until a white solid precipitated. The mixture was subjected to filtration, and the filter cake was washed with ethanol and dried in a vacuum to obtain a white solid. Yield 58%, mp 170–172 °C. ^1^H NMR (CDCl_3_) δ: 7.14–7.19 (4H, m), 7.09 (1H, t, J_1_ = 8.0 Hz, J_2_ = 8.0 Hz), 5.37–5.41 (1H, m), 4.37–4.91 (3H, m), 3.47–4.00 (3H, m), 2.82–3.01 (2H, m), 1.44 (9H, s). ^13^C NMR (CDCl_3_) δ: 28.2 (3C), 33.2, 38.3, 47.5, 50.2, 56.5, 80.3, 118.1, 126.2, 127.8 (2C), 129.2 (2C), 136.9, 144.1, 152.8, 155.4, 171.5. ESI-MS (*m*/*z*): calculated for C20H23F3N5O3, [M-H]^+^: 438.44; found 438.03.

### 3.8. Synthesis of Tert-Butyl (S)-(3-(1H-indol-3-yl)-1-oxo-1-(3-(trifluoromethyl)-5,6-dihydro-[1,2,4]triazolo[4,3-a]pyrazin-7(8H)-yl)propan-2-yl)carbamate (***VIIb***)

The synthesis method was the same as that in Section 3.7, except that the raw material Boc-L-phenylalanine was replaced by Boc-L-tryptophan to obtain a white solid. Yield 43%, mp 229–232 °C. ^1^H NMR (DMSO-d6) δ: 10.86 (1H, s), 7.42 (1H, t, J_1_ = 8.0 Hz, J_2_ = 8.0 Hz), 7.12–7.23 (2H, m), 6.84–6.96 (2H, m), 4.63–4.75 (3H, m), 3.72–4.07 (3H, m), 2.94–3.20 (3H, m), 1.35 (9H, s). ^13^C NMR (DMSO-d6) δ: 28.0 (3C), 28.7, 33.2, 47.7, 49.8, 57.5, 80.1, 109.1, 112.5, 118.0, 118.9, 120.4, 122.5, 125.1, 127.6, 137.0, 143.9, 153.0, 155.6, 171.4. ESI-MS (*m*/*z*): calculated for C22H26F3N6O3, [M+H]^+^: 479.48; found 479.34.

### 3.9. (S)-2-amino-3-phenyl-1-(3-(trifluoromethyl)-5,6-dihydro-[1,2,4]triazolo[4,3-a]pyrazin-7(8H)-yl)propan-1-one (***VIIIa***) or (S)-2-amino-3-(1H-indol-3-yl)-1-(3-(trifluoromethyl)-5,6-dihydro-[1,2,4]triazolo[4,3-a]pyrazin-7(8H)-yl)propan-1-one (***VIIIb***)

The intermediate **VIIa** or **VIIb** (2.0 g) was placed in a 50 mL flask, DCM (20 mL) and trifluoroacetic acid (20 mL) were added, and the mixture was stirred at room temperature for 4 h. The solvent was then evaporated under reduced pressure, and saturated aqueous Na_2_CO_3_ was added slowly. After stirring at room temperature for 1 h, the mixture was subjected to extraction with DCM, dried over anhydrous Na_2_SO_4_, and the organic phase was concentrated to obtain crude **VIIIa** or **VIIIb**, which was directly used in the following reaction.

### 3.10. Generalized Procedure for the Synthesis of Compound ***1***

Intermediate **VIIIa** or **VIIIb** (0.50 mmol) was placed in a 25 mL flask, methanol (15 mL) was added, and stirred to dissolve the intermediate. The solution obtained was cooled on ice, an aldehyde (0.42 mmol), NaBH_3_CN (151 mg, 0.89 mmol), and glacial acetic acid (38 mg, 0.62 mmol) were added, and the reaction was carried out overnight at room temperature. The solvent was then evaporated under reduced pressure, saturated aqueous Na_2_CO_3_ was added, followed by extraction with ethyl acetate to separate the organic phase. The organic phase was then dried with anhydrous Na_2_SO_4_ and filtered, and the filtrate was concentrated under reduced pressure. The residue was subjected to silica gel column chromatography.

(*S*)-2-(butylamino)-3-phenyl-1-(3-(trifluoromethyl)-5,6-dihydro-[1,2,4]triazolo[4,3-*a*]pyrazin-7(8*H*)-yl)propan-1-one (**1a**)

Purified by column chromatography (Ethyl acetate/cyclohexane, 20/80); white solid; yield 69%, mp 89–91 °C. ^1^H NMR (DMSO-d6) δ: 7.36–7.40 (2H, m), 7.08–7.16 (3H, m), 4.44 (2H, s), 3.94–4.00 (4H, m), 3.35–3.38 (3H, m), 2.85–2.94 (2H, m), 1.55–1.58 (2H, m), 1.35–1.39 (2H, m), 0.93 (3H, t). ^13^C NMR (DMSO-d6) δ: 13.9, 19.6, 30.8, 33.6, 40.6, 46.5, 47.2, 49.6, 62.6, 118.5 126.3, 128.6 (2C), 129.6 (2C), 136.6, 143.5, 152.8, 169.6. ESI-MS (*m*/*z*): calculated for [M+H]^+^: 396.43; found 396.30. Elemental analysis for C19H24F3N5O. Calculated (%): C, 57.71; H, 6.12; N, 17.71. Found (%): C, 57.78; H, 6.15; N, 17.63.

(*S*)-2-(benzylamino)-3-phenyl-1-(3-(trifluoromethyl)-5,6-dihydro-[1,2,4]triazolo[4,3-*a*]pyrazin-7(8*H*)-yl)propan-1-one (**1b**)

Purified by column chromatography (Ethyl acetate/cyclohexane, 20/80); white solid; yield 56%, mp 120–122 °C. ^1^H NMR (DMSO-d6) δ: 7.54–7.64 (3H, m), 7.42–7.43 (2H, m), 7.00–7.17 (5H, m), 4.56 (2H, s), 4.14–4.27 (3H, m), 3.61–3.94 (4H, m), 3.14–3.24 (2H, m). ^13^C NMR (DMSO-d6) δ: 33.6, 41.5, 46.2, 48.6, 52.5, 59.6, 118.4, 126.8, 127.3, 127.9 (2C), 128.3 (2C), 128.8 (2C), 129.9 (2C), 143.9, 152.2, 168.3. ESI-MS (*m*/*z*): calculated for [M+H]^+^: 430.45; found 430.15. Elemental analysis for C22H22F3N5O. Calculated (%): C, 61.53; H, 5.16; N, 16.31. Found (%): C, 61.57; H, 5.11; N, 16.37.

(*S*)-2-((furan-2-ylmethyl)amino)-3-phenyl-1-(3-(trifluoromethyl)-5,6-dihydro-[1,2,4]triazolo[4,3-*a*]pyrazin-7(8*H*)-yl)propan-1-one (**1c**)

Purified by column chromatography (Ethyl acetate/cyclohexane, 20/80); white solid; yield 71%, mp 75–77 °C. ^1^H NMR (DMSO-d6) δ: 7.01–7.35 (6H, m), 6.25–6.51 (2H, m), 4.10–4.38 (2H, m), 3.11–3.83 (7H, m), 2.85–3.04 (2H, m). ^13^C NMR (DMSO-d6) δ: 33.0, 38.4, 46.7, 47.2, 49.3, 59.6, 110.1, 110.9, 118.0, 126.7, 128.4 (2C), 129.3 (2C), 138.6, 143.1, 143.5, 146.8, 152.5, 170.7. ESI-MS (*m*/*z*): calculated for [M+H]^+^: 420.41; found 420.28. Elemental analysis for C20H20F3N5O2. Calculated (%): C, 57.28; H, 4.81; N, 16.70. Found (%): C, 57.19; H, 4.85; N, 16.78.

(*S*)-3-phenyl-1-(3-(trifluoromethyl)-5,6-dihydro-[1,2,4]triazolo[4,3-*a*]pyrazin-7(8*H*)-yl)-2-((3,4,5-trimethoxybenzyl)amino)propan-1-one (**1d**)

Purified by column chromatography (Ethyl acetate/cyclohexane, 20/80); white solid; yield 64%, mp 58–61 °C. ^1^H NMR (DMSO-d6) δ: 6.90–7.14 (5H, m), 6.50 (2H, s), 4.27–4.37 (2H, m), 3.89–3.94 (2H, m), 3.85 (9H, s), 3.55–3.70 (3H, m), 3.19–3.43 (2H, m), 2.72–3.09 (2H, m). ^13^C NMR (DMSO-d6) δ: 33.5, 40.2, 47.4, 49.5, 52.3, 56.3 (2C), 61.1, 63.5, 103.2 (2C), 118.2, 126.8, 128.3 (2C), 129.2 (2C), 131.4, 138.2, 142.3, 153.7 (2C), 143.8, 152.4, 172.2. ESI-MS (*m*/*z*): calculated for [M+H]^+^: 520.53; found 520.25. Elemental analysis for C25H28F3N5O4. Calculated (%): C, 57.80; H, 5.43; N, 13.48. Found (%): C, 57.88; H, 5.37; N, 13.43.

(*S*)-2-((4-bromobenzyl)amino)-3-phenyl-1-(3-(trifluoromethyl)-5,6-dihydro-[1,2,4]triazolo[4,3-*a*]pyrazin-7(8*H*)-yl)propan-1-one (**1e**)

Purified by column chromatography (Ethyl acetate/cyclohexane, 20/80); white solid; yield 76%, mp 141–142 °C. ^1^H NMR (CDCl_3_) δ: 7.40 (2H, d, J = 8.5 Hz), 7.16–7.19 (3H, m), 7.04–7.09 (3H, m), 6.94 (1H, t, J1 = 7.0 Hz, J2 = 7.0 Hz), 4.27–4.38 (2H, m), 3.58–3.91 (5H, m), 3.29–3.42 (2H, m), 2.72–3.07 (2H, m). ^13^C NMR (CDCl_3_) δ: 33.5, 40.8, 48.1, 49.7, 50.3, 60.7, 118.5, 119.7, 126.9, 128.4 (2C), 129.6 (2C), 130.8 (2C), 131.3 (2C), 138.6, 139.7, 143.9, 152.7, 170.3. ESI-MS (*m*/*z*): calculated for [M+2]^+^: 510.34; found 510.27. Elemental analysis for C22H21BrF3N5O. Calculated (%): C, 51.98; H, 4.16; N, 13.78. Found (%): C, 51.87; H, 4.21; N, 13.86.

(*S*)-2-(butylamino)-3-(1*H*-indol-3-yl)-1-(3-(trifluoromethyl)-5,6-dihydro-[1,2,4]triazolo[4,3-*a*]pyrazin-7(8*H*)-yl)propan-1-one (**1f**)

Purified by column chromatography (Ethyl acetate/cyclohexane, 20/80); white solid; yield 53%, mp 151–153 °C. ^1^H NMR (CDCl_3_) δ: 8.03 (1H, s), 7.47 (1H, d, J = 8.5 Hz), 6.98–7.16 (4H, m), 4.33–4.66 (3H, m), 3.63–3.89 (2H, m), 2.86–3.31 (4H, m), 2.51–2.54 (2H, t), 1.46–1.52 (2H, m), 1.33–1.39 (2H, m), 0.93 (3H, t). ^13^C NMR (CDCl_3_) δ: 13.6, 19.7, 29.3, 30.9, 33.4, 47.6, 48.0, 49.6, 63.3, 109.2, 112.5, 118.1, 119.2, 119.8, 120.6, 123.0, 127.6, 137.2, 143.6, 152.3, 171.1. ESI-MS (*m*/*z*): calculated for [M+H]^+^: 435.47; found 435.48. Elemental analysis for C21H25F3N6O. Calculated (%): C, 58.06; H, 5.80; N, 19.34. Found (%): C, 58.17; H, 5.76; N, 19.26.

(*S*)-2-(benzylamino)-3-(1*H*-indol-3-yl)-1-(3-(trifluoromethyl)-5,6-dihydro-[1,2,4]triazolo[4,3-*a*]pyrazin-7(8*H*)-yl)propan-1-one (**1g**)

Purified by column chromatography (Ethyl acetate/cyclohexane, 30/70); white solid; yield 57%, mp 123–126 °C. ^1^H NMR (CDCl_3_) δ: 8.45 (1H, s), 7.44 (1H, d, J = 8.5 Hz), 7.23–7.32 (5H, m), 7.16 (1H, s), 6.96–7.04 (3H, m), 4.12–4.62 (3H, m), 3.74–3.91 (3H, m), 3.26–3.56 (2H, m), 2.80–3.09 (3H, m). ^13^C NMR (CDCl_3_) δ: 30.6, 33.4, 47.6, 49.8, 51.7, 63.2, 109.1, 112.6, 118.2, 119.1, 120.5, 122.8, 124.8, 126.6, 127.6, 128.7 (2C), 129.4 (2C), 137.1, 140.5, 143.7, 152.8, 170.9. ESI-MS (*m*/*z*): calculated for [M+H]^+^: 469.48; found 469.40. Elemental analysis for C24H23F3N6O. Calculated (%): C, 61.53; H, 4.95; N, 17.94. Found (%): C, 61.62; H, 4.91; N, 17.76.

(*S*)-2-((furan-2-ylmethyl)amino)-3-(1*H*-indol-3-yl)-1-(3-(trifluoromethyl)-5,6-dihydro-[1,2,4]triazolo[4,3-*a*]pyrazin-7(8*H*)-yl)propan-1-one (**1h**)

Purified by column chromatography (Ethyl acetate/cyclohexane, 30/70); white solid; yield 63%, mp 110–112 °C. ^1^H NMR (CDCl_3_) δ: 8.14 (1H, s), 7.45 (1H, d, J = 8.5 Hz), 7.33 (1H, s), 6.97–7.07 (4H, m), 6.27 (1H, m), 6.16 (1H, t, J1 = 8.5 Hz, J2 = 3.0 Hz), 4.36–4.48 (2H, m), 4.19–4.23 (1H, m), 3.59–3.88 (4H, m), 3.23–3.28 (1H, m), 2.81–3.12 (3H, m). ^13^C NMR (CDCl_3_) δ: 29.6, 33.0, 47.4, 48.1, 49.5, 59.8, 109.1, 110.3, 111.2, 112.6, 118.0, 119.5, 120.6, 122.8, 125.6, 127.5, 137.1, 142.8, 143.4, 146.5, 152.3, 170.5. ESI-MS (*m*/*z*): calculated for [M+H]^+^: 459.45; found 459.21. Elemental analysis for C22H21F3N6O2. Calculated (%): C, 57.64; H, 4.62; N, 18.33. Found (%): C, 57.72; H, 4.56; N, 18.21.

(*S*)-3-(1*H*-indol-3-yl)-1-(3-(trifluoromethyl)-5,6-dihydro-[1,2,4]triazolo[4,3-*a*]pyrazin-7(8*H*)-yl)-2-((3,4,5-trimethoxybenzyl)amino)propan-1-one (**1i**)

Purified by column chromatography (Ethyl acetate/cyclohexane, 30/70); white solid; yield 62%, mp 83–85 °C. ^1^H NMR (DMSO-d6) δ: 8.28 (1H, s), 7.45 (1H, d, J = 8.5 Hz), 7.13 (1H, s), 7.00–7.06 (3H, m), 6.58 (2H, s), 4.43–4.63 (2H, m), 4.06–4.15 (1H, m), 3.86 (9H, s), 3.58–3.75 (3H, m), 3.27–3.48 (2H, m), 2.79–3.14 (3H, m). ^13^C NMR (DMSO-d6) δ: 30.7, 33.3, 47.8, 49.8, 51.5, 56.2 (2C), 60.9, 62.8, 105.2 (2C), 108.6, 112.4, 118.3, 119.2, 120.5, 122.8, 125.2, 127.6, 131.2, 136.8, 137.1, 152.7 (2C), 143.8, 152.6, 170.7. ESI-MS (*m*/*z*): calculated for [M+H]^+^: 559.56; found 559.78. Elemental analysis for C27H29F3N6O4. Calculated (%): C, 58.06; H, 5.23; N, 15.05. Found (%): C, 58.22; H, 5.21; N, 15.01.

(*S*)-2-((4-bromobenzyl)amino)-3-(1*H*-indol-3-yl)-1-(3-(trifluoromethyl)-5,6-dihydro-[1,2,4]triazolo[4,3-*a*]pyrazin-7(8*H*)-yl)propan-1-one (**1j**)

Purified by column chromatography (Ethyl acetate/cyclohexane, 30/70); white solid; yield 65%, mp 106–108 °C. ^1^H NMR (DMSO-d6) δ: 7.82–7.84 (2H, d, J = 8.5 Hz), 7.30–7.33 (2H, d, J = 8.5 Hz), 6.96–7.32 (5H, m), 4.50 (2H, s), 3.98–4.22 (5H, m), 3.80–3.82 (2H, m), 2.93–3.06 (2H, m). ^13^C NMR (DMSO-d6) δ: 30.8, 33.2, 47.6, 49.7, 51.5, 64.2, 109.4, 112.5, 118.1, 119.3, 120.6, 122.1, 122.5, 125.4, 127.6, 130.3 (2C), 131.9 (2C), 136.9, 138.9, 143.6, 152.4, 172.1. ESI-MS (*m*/*z*): calculated for [M]^+^: 547.38; found 547.16. Elemental analysis for C24H22BrF3N6O. Calculated (%): C, 52.66; H, 4.05; N, 15.35. Found (%): C, 52.82; H, 4.01; N, 15.27.

### 3.11. Generalized Procedure for the Synthesis of Compound ***2***

Intermediate **VIIIa** or **VIIIb** (0.44 mmol) was placed in a 25 mL flask, DCM (10 mL) was added, and stirred to dissolve the intermediate. Triethylamine (45 mg, 0.44 mmol) was added, and the mixture was cooled to 0 °C on ice. Then, sulfonyl chloride (0.40 mmol) was slowly added dropwise using a constant-pressure dropping funnel, and the reaction was carried out for 3 h. The reaction was quenched by the addition of saturated aqueous Na_2_CO_3_, and the mixture was subjected to dilution and extraction with DCM. The organic phase was thus separated, dried with anhydrous Na_2_SO_4_, and filtered. The filtrate was concentrated under reduced pressure, and the residue was subjected to silica gel column chromatography.

(*S*)-*N*-(1-oxo-3-phenyl-1-(3-(trifluoromethyl)-5,6-dihydro-[1,2,4]triazolo[4,3-*a*]pyrazin-7(8*H*)-yl)propan-2-yl)methanesulfonamide (**2a**)

Purified by column chromatography (Ethyl acetate/cyclohexane, 40/60); white solid; yield 73%, mp 123–125 °C. ^1^H NMR (DMSO-d6) δ: 7.88 (1H, s), 6.96–7.28 (5H, m), 4.96–5.01 (1H, m), 4.62–4.79 (2H, m), 3.98–4.20 (2H, m), 3.39–3.54 (2H, m), 2.85–2.97 (2H, m), 2.81 (3H, s). ^13^C NMR (DMSO-d6) δ: 33.4, 37.6, 43.1, 47.7, 50.0, 58.5, 118.6, 126.7, 127.9 (2C), 129.4 (2C), 136.8, 143.9, 152.8, 171.7. ESI-MS (*m*/*z*): calculated for [M-H]^+^: 416.41; found 416.08. Elemental analysis for C16H18F3N5O3S. Calculated (%): C, 46.04; H, 4.35; N, 16.78. Found (%): C, 46.22; H, 4.31; N, 16.67.

(*S*)-*N*-(1-oxo-3-phenyl-1-(3-(trifluoromethyl)-5,6-dihydro-[1,2,4]triazolo[4,3-*a*]pyrazin-7(8*H*)-yl)propan-2-yl)benzenesulfonamide (**2b**)

Purified by column chromatography (Ethyl acetate/cyclohexane, 40/60); white solid; yield 77%, mp. 170–173 °C. ^1^H NMR (DMSO-d6) δ: 8.51 (1H, m), 7.42–7.72 (5H, m), 7.12–7.15 (3H, m), 7.01 (1H, m), 4.69–4.73 (2H, m), 4.52–4.54 (1H, m), 3.68–4.05 (3H, m), 3.20–3.38 (2H, m), 2.78–2.80 (1H, m). ^13^C NMR (DMSO-d6) δ: 33.5, 37.8, 47.5, 49.7, 56.7, 118.5, 126.5, 128.4 (2C), 128.9 (2C), 129.5 (2C), 130.1 (2C), 131.7, 138.5, 144.2, 143.5, 152.5, 171.3. ESI-MS (*m*/*z*): calculated for [M-H]^+^: 478.48; found 478.04. Elemental analysis for C21H20F3N5O3S. Calculated (%): C, 52.61; H, 4.20; N, 14.61. Found (%): C, 52.56; H, 4.23; N, 14.69.

(*S*)-*N*-(1-oxo-3-phenyl-1-(3-(trifluoromethyl)-5,6-dihydro-[1,2,4]triazolo[4,3-*a*]pyrazin-7(8*H*)-yl)propan-2-yl)isoquinoline-5-sulfonamide (**2c**)

Purified by column chromatography (Ethyl acetate/cyclohexane, 40/60); white solid; yield 72%, mp 242–244 °C. ^1^H NMR (DMSO-d6) δ: 9.40 (1H, s), 8.62–8.64 (1H, m), 8.16–8.42 (3H, m), 7.62–7.65 (1H, m), 6.85–7.07 (5H, m), 4.51–4.74 (3H, m), 3.80–4.03 (2H, m), 3.46–3.68 (2H, m), 2.69–2.88 (2H, m). ^13^C NMR (DMSO-d6) δ: 33.4, 38.7, 47.7, 49.8, 55.7, 118.3, 119.5, 126.6, 127.2, 127.8, 128.4 (2C), 129.1, 129.3 (2C), 130.2, 131.7, 138.4, 141.2, 143.7, 145.6, 152.7, 153.2, 170.9. ESI-MS (*m*/*z*): calculated for [M+H]^+^: 531.53; found 531.38. Elemental analysis for C24H21F3N6O3S. Calculated (%): C, 54.34; H, 3.99; N, 15.84. Found (%): C, 54.21; H, 4.03; N, 15.92.

(*S*)-*N*-(3-(1*H*-indol-3-yl)-1-oxo-1-(3-(trifluoromethyl)-5,6-dihydro-[1,2,4]triazolo[4,3-*a*]pyrazin-7(8*H*)-yl)propan-2-yl)methanesulfonamide (**2d**)

Purified by column chromatography (Ethyl acetate/cyclohexane, 40/60); white solid; yield 68%, mp 98–101 °C. ^1^H NMR (DMSO-d6) δ: 8.18 (1H, s), 7.52 (1H, d, J = 8.5 Hz), 7.01–7.23 (4H, m), 4.37–4.61 (2H, m), 3.86–4.12 (2H, m), 3.16–3.53 (3H, m), 2.82–2.96 (2H, m). ^13^C NMR (DMSO-d6) δ: 31.2, 33.5, 43.3, 47.9, 50.1, 57.6, 108.9, 112.4, 118.3, 119.1, 120.6, 122.4, 125.3, 127.6, 137.2, 143.8, 153.1, 171.6. ESI-MS (*m*/*z*): calculated for [M+H]^+^: 457.44; found 457.33. Elemental analysis for C18H19F3N6O3S. Calculated (%): C, 47.37; H, 4.20; N, 18.41. Found (%): C, 47.29; H, 4.16; N, 18.56.

(*S*)-*N*-(3-(1*H*-indol-3-yl)-1-oxo-1-(3-(trifluoromethyl)-5,6-dihydro-[1,2,4]triazolo[4,3-*a*]pyrazin-7(8*H*)-yl)propan-2-yl)benzenesulfonamide (**2e**)

Purified by column chromatography (Ethyl acetate/cyclohexane, 40/60); white solid; yield 65%, mp 144–146 °C. ^1^H NMR (DMSO-d6) δ: 8.38 (1H, s), 7.70–7.71 (2H, d, J = 8.5 Hz), 7.49–7.54 (3H, m), 7.16–7.48 (5H, m), 4.54 (2H, s), 3.68–3.95 (5H, m), 2.94–3.05 (2H, m). ^13^C NMR (DMSO-d6) δ: 31.1, 33.3, 48.6, 50.7, 57.8, 108.6, 112.1, 118.1, 120.1, 121.6, 122.3, 124.8, 126.5 (2C), 127.8, 129.2 (2C), 132.7, 137.4, 143.6, 152.8, 172.6. ESI-MS (*m*/*z*): calculated for [M+H]^+^: 519.52; found 519.30. Elemental analysis for C23H21F3N6O3S. Calculated (%): C, 53.28; H, 4.08; N, 16.21. Found (%): C, 53.45; H, 4.11; N, 16.17.

### 3.12. Antibacterial Activity Evaluation In Vitro

The microbroth dilution method was used to evaluate the in vitro antibacterial activities of all the synthesized compounds [44,45]. Gram-positive bacteria *S. aureus* and Gram-negative bacteria *E. coli* were selected as the tested organisms. Typically, the tested compounds were dissolved in dimethylformamide to form a stock solution (512 μg/mL), and the gradient concentrations ranged from 256 to 4 μg/mL were obtained via two-fold serial dilution with distilled water. The sample solutions with varied concentrations were filtrated through a 0.22 μm Millipore filter and transferred to the wells of a sterilized 96-well microplate (100 uL per well). After freeze-drying, the microplate was sealed for use. Ampicillin, a marketed drug, was selected as a positive control. The tested bacteria grown to mid-log phase were collected and adjusted to a turbidity of a 0.5 McFarland standard (approximate 1.0 × 10^8^ CFU/mL). The bacterial suspension was diluted 1000-fold to approximately 10^5^ CFU/mL with the sterilized Mueller–Hinton broth, and then 100 μL of bacterial suspension per well was inoculated into the pre-treated 96-well plates containing the compounds and incubated at 37 °C for 24 h. The lowest concentration value of a sample for visible growth of the tested bacteria was recorded as the MIC of the sample. Three parallel experiments were conducted for each tested compound, with a blank control group and a positive control group.

## 4. Conclusions

Using 3-(trifluoromethyl)-1,2,4-triazolo[4,3-*a*]pyrazine as the key scaffold, some novel triazolo[4,3-*a*]pyrazine derivatives have been synthesized in appreciable yields through two different approaches and characterized using various techniques, such as melting point, ^1^H and ^13^C NMR spectroscopy, MS, and elemental analysis. All the newly synthesized compounds were screened for antibacterial activity using microbroth dilution. A majority of compounds have emerged as effective inhibitory agents against Gram-positive bacteria *S. aureus* and Gram-negative bacteria *E. coli*. Among all the tested compounds, compound **2e** exhibited superior antibacterial activity (MICs: 32 μg/mL against *S. aureus* and 16 μg/mL against *E. coli*) and was comparable to the first-line antimicrobial agent ampicillin, and thus can be further optimized according to the structure–activity relationship.

## Figures and Tables

**Table 1:** The synthesized triazolo[4,3-*a*]pyrazine derivatives **1** and **2**.

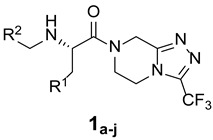 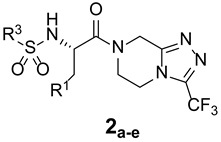				
Compound	R^1^	R^2^	R^3^	Yield (%) ^#^
**1a**	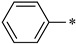	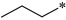	**_**	69
**1b**	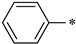	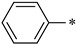	**_**	56
**1c**	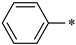	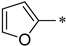	**_**	71
**1d**	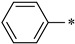	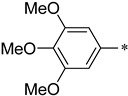	**_**	64
**1e**	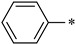	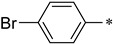	**_**	76
**1f**	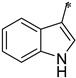	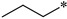	**_**	53
**1g**	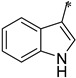	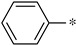	**_**	57
**1h**	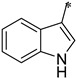	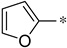	**_**	63
**1i**	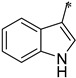	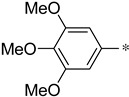	**_**	62
**1j**	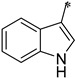	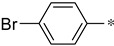	**_**	65
**2a**	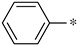	**_**	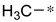	73
**2b**	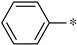	**_**	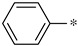	77
**2c**	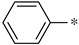	**_**	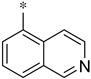	72
**2d**	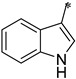	**_**	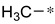	68
**2e**	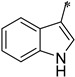	**_**	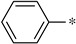	65

* substituted site; ^#^ yield of the isolation stage.

**Table 2:** In vitro antibacterial activity of synthesized triazolo[4,3-*a*]pyrazine derivatives **1** and **2**.

Compound	MIC (μg/mL)	
	*S. aureus*	*E. coli*
Ampicillin	32	8
**1a**	64	64
**1b**	>256	256
**1c**	>256	>256
**1d**	128	64
**1e**	128	128
**1f**	64	32
**1g**	128	64
**1h**	256	128
**1i**	64	32
**1j**	128	64
**2a**	256	>256
**2b**	128	64
**2c**	64	64
**2d**	256	128
**2e**	32	16

## Data Availability

Data is contained within the article.

## Supplementary Materials

The following supporting information can be downloaded at: https://www.mdpi.com/article/10.3390/molecules28237876/s1, ^1^H NMR and ^13^C NMR spectra (Figures S1–S8) of some of the novel compounds and mass spectra (Figures S9–S23) of all the compounds.
