# Supplementary material for: Synthesis and Antibacterial Activity of Novel Triazolo[4,3-a]pyrazine Derivatives
Source: Molecules. 2023 Nov 30;28(23):7876. doi: 10.3390/molecules28237876 (PMC10708386; doi:10.3390/molecules28237876)
Supplement: Supplementary file 1 [file molecules-28-07876-s001.zip › molecules-2697118-supplementary.pdf]

Supplementary Information

Synthesis and Antibacterial Activity of Novel Triazolo[4,3-a]pyrazine Derivatives

Zhang Hu, Hongrui Dong, Zhenyu Si, Yurong Zhao and Yuanwei Liang *

FacFaculty of Chemistry and Environmental Science, Guangdong Ocean University, Zhanjiang 524088, China; hu-zhang@gdou.edu.cn (Z.H.)

* Correspondence: liangyw@gdou.edu.cn

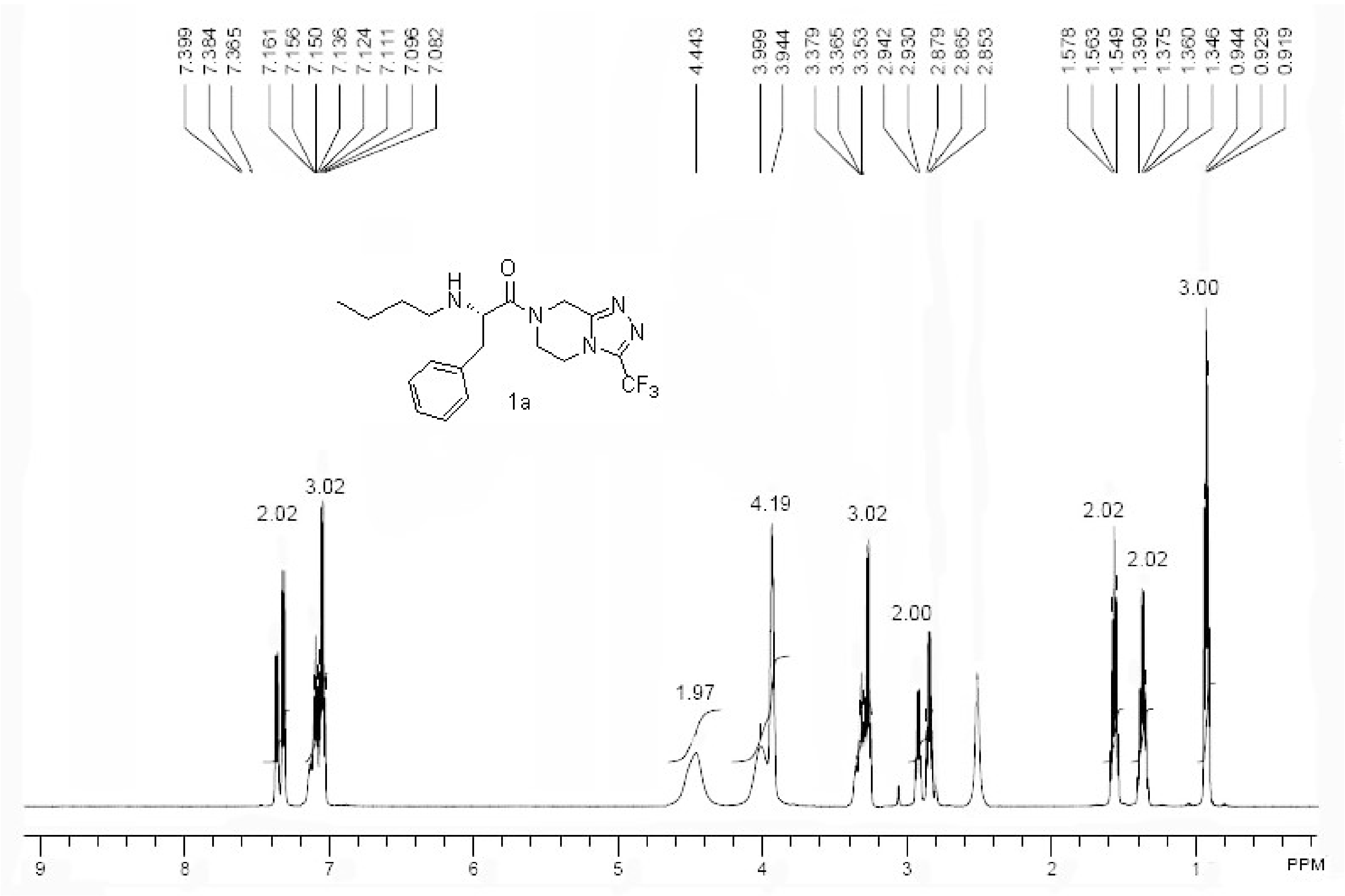

Figure S1. ^{1}H NMR spectrum of compound **1a**.

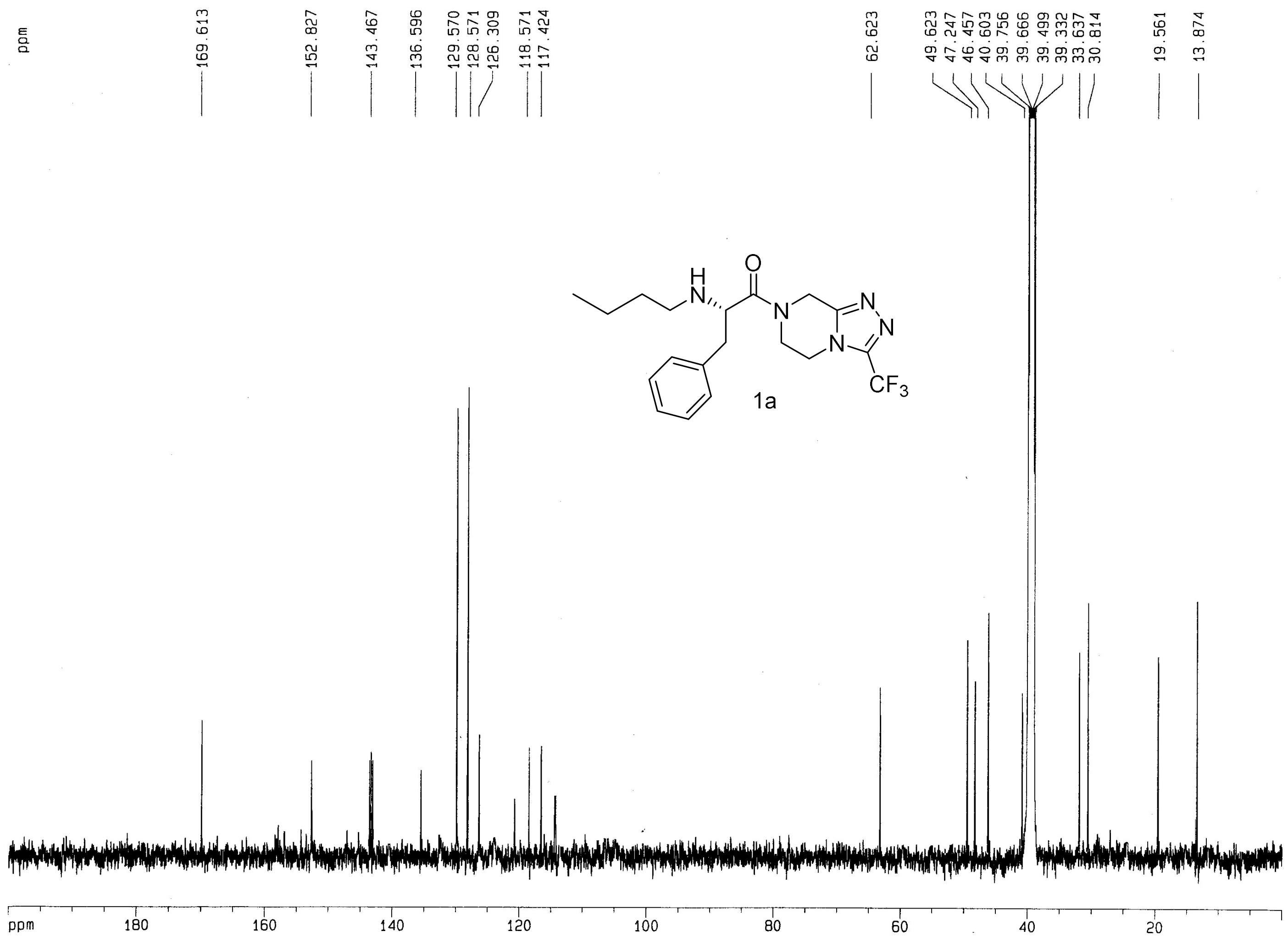

Figure S2. ^{13}C NMR spectrum of compound **1a**.

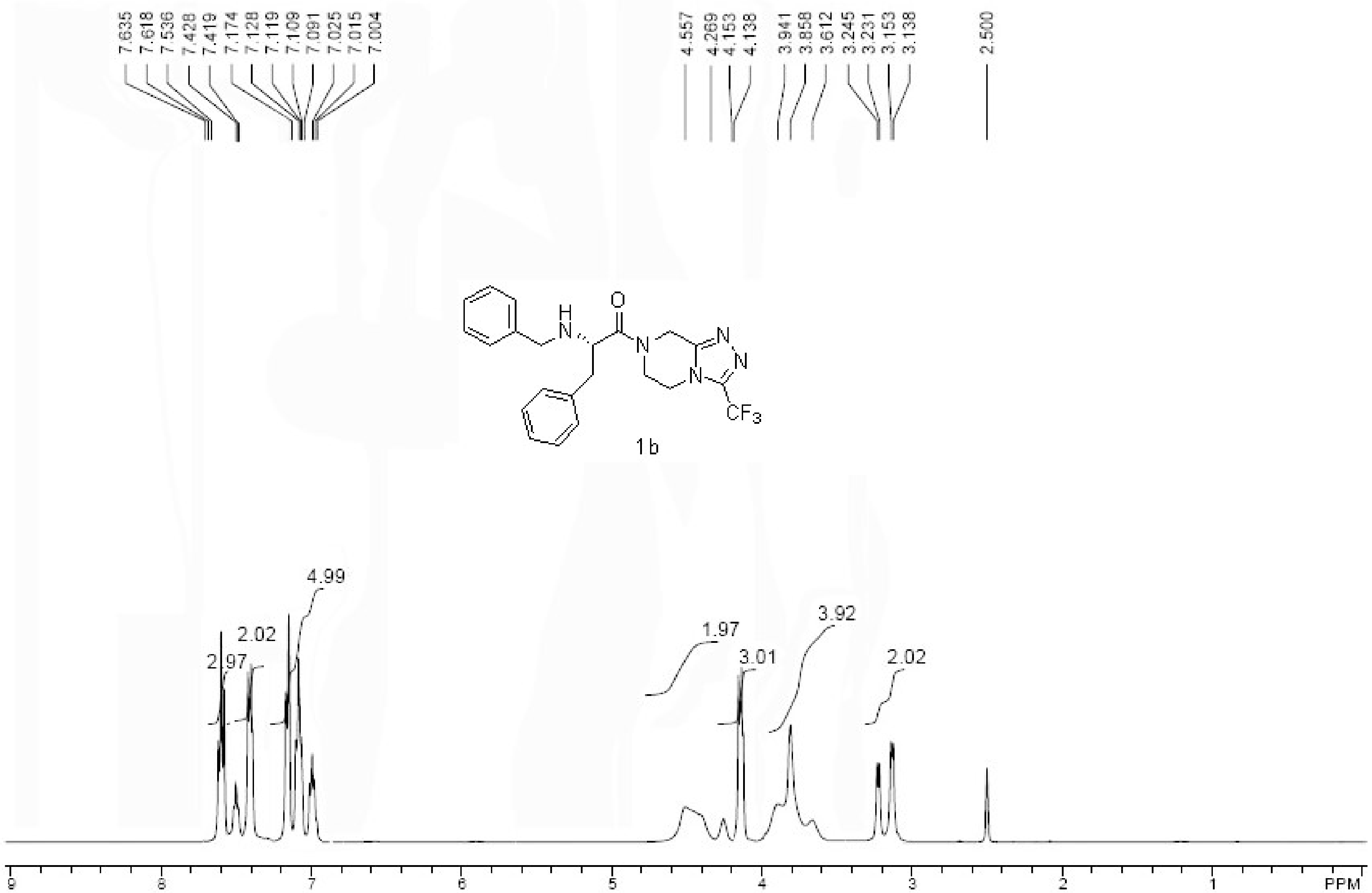

Figure S3. ^{1}H NMR spectrum of compound **1b**.

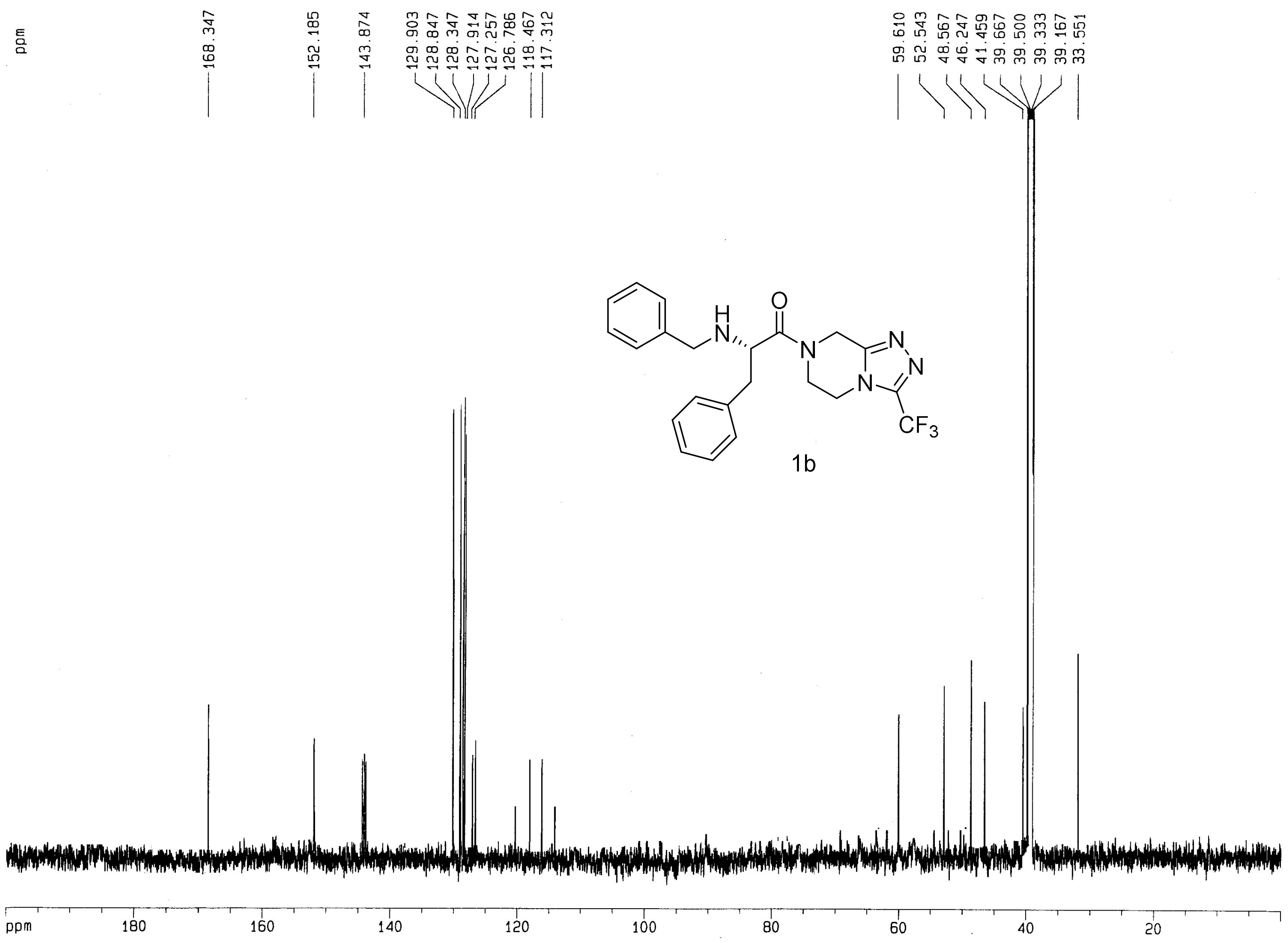

Figure S4. ^{13}C NMR spectrum of compound **1b**.

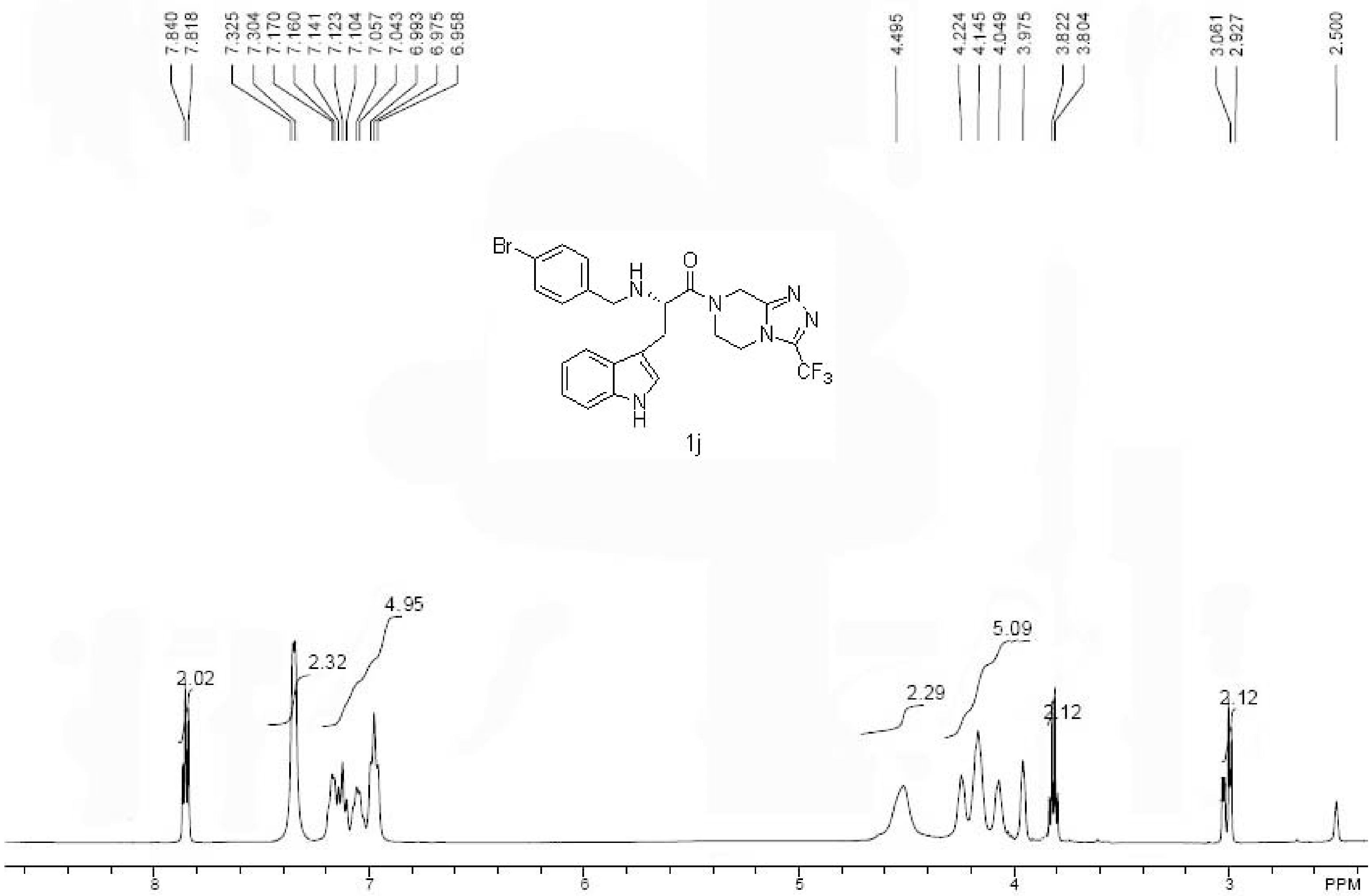

Figure S5. ^{1}H NMR spectrum of compound **1j**.

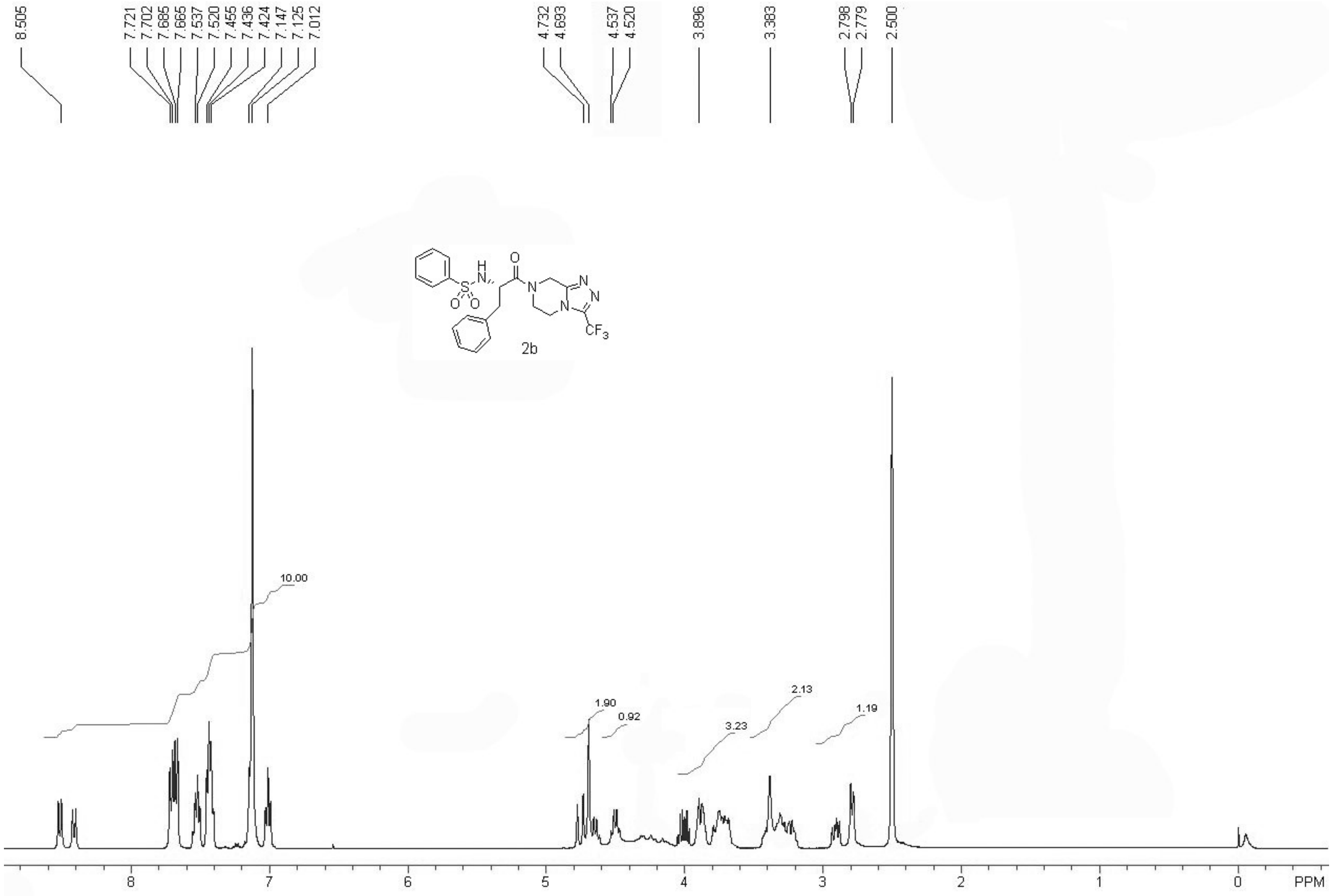

Figure S6. ^{1}H NMR spectrum of compound **2b**.

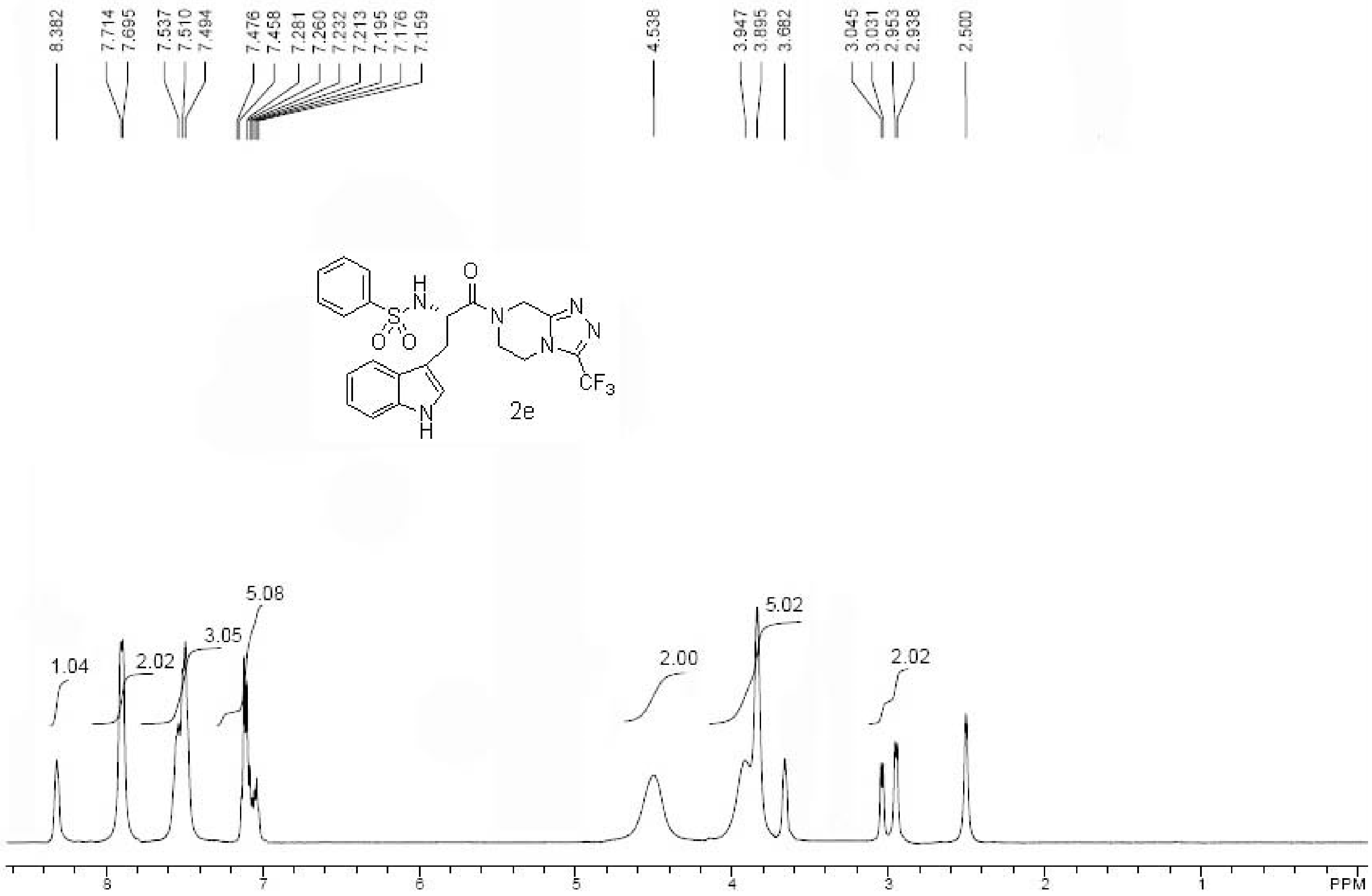

Figure S7. ^{1}H NMR spectrum of compound **2e**.

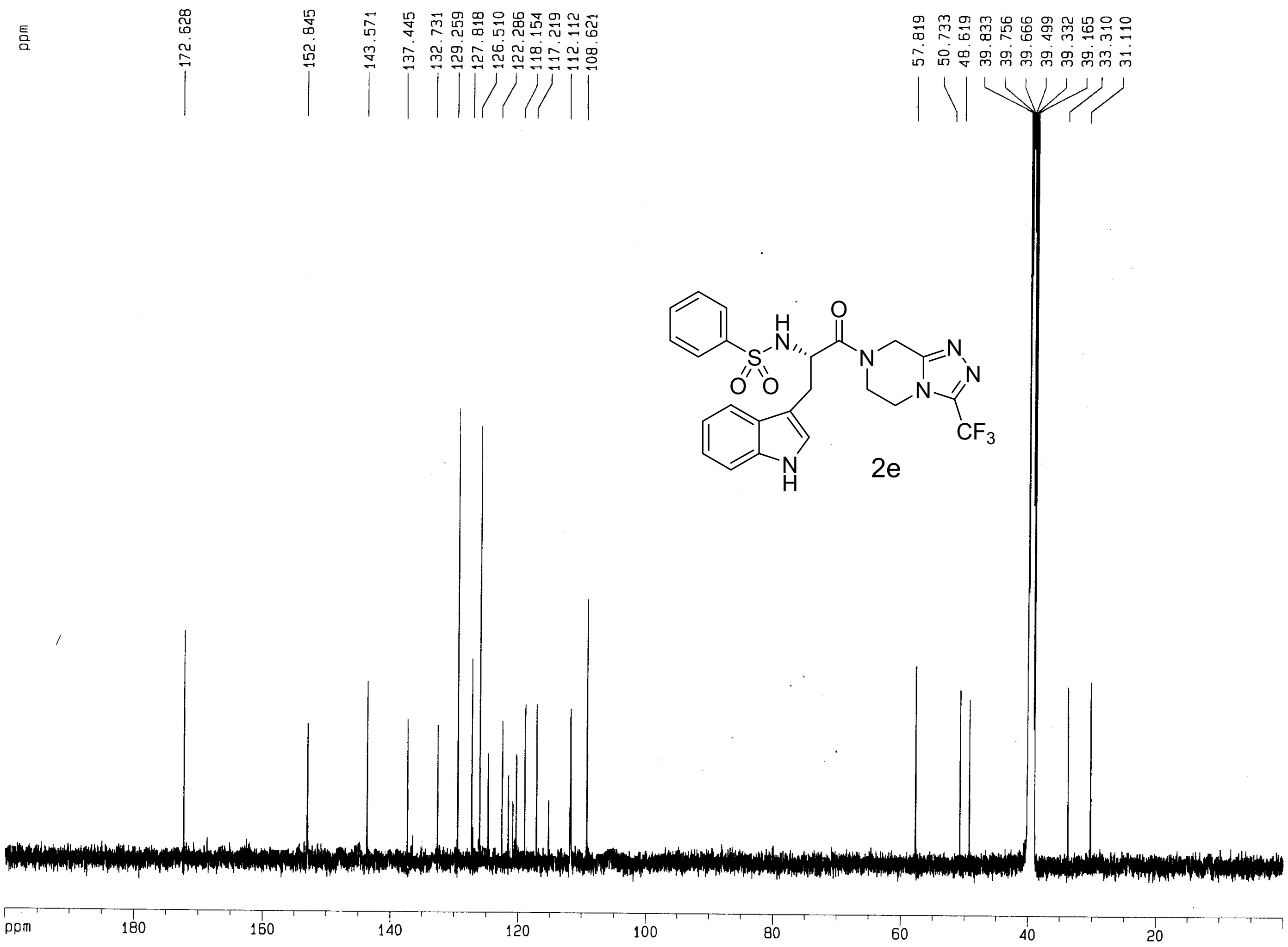

Figure S8. ^{13}C NMR spectrum of compound **2e**.

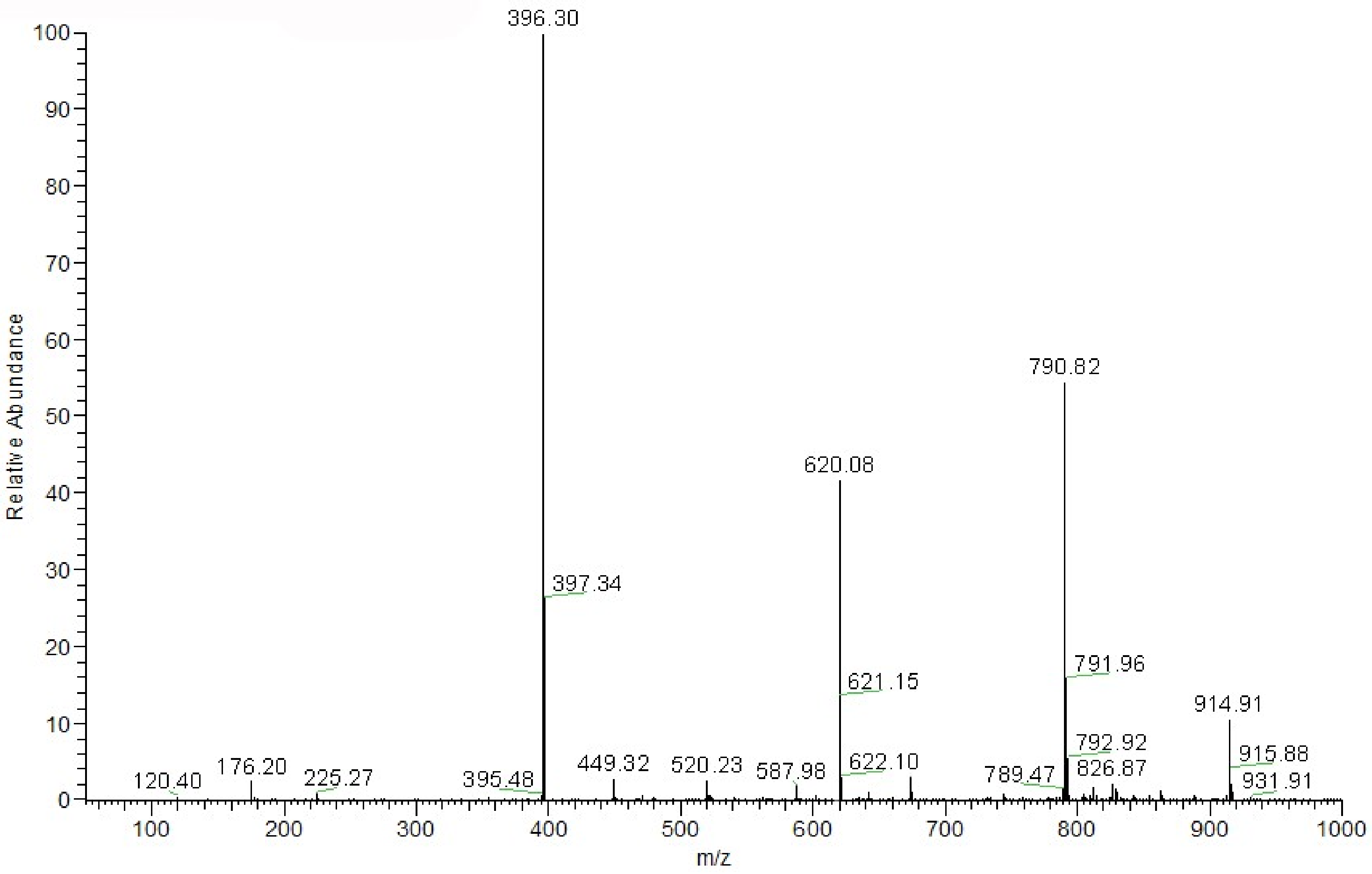

Figure S9. Mass spectra of compound **1a**.

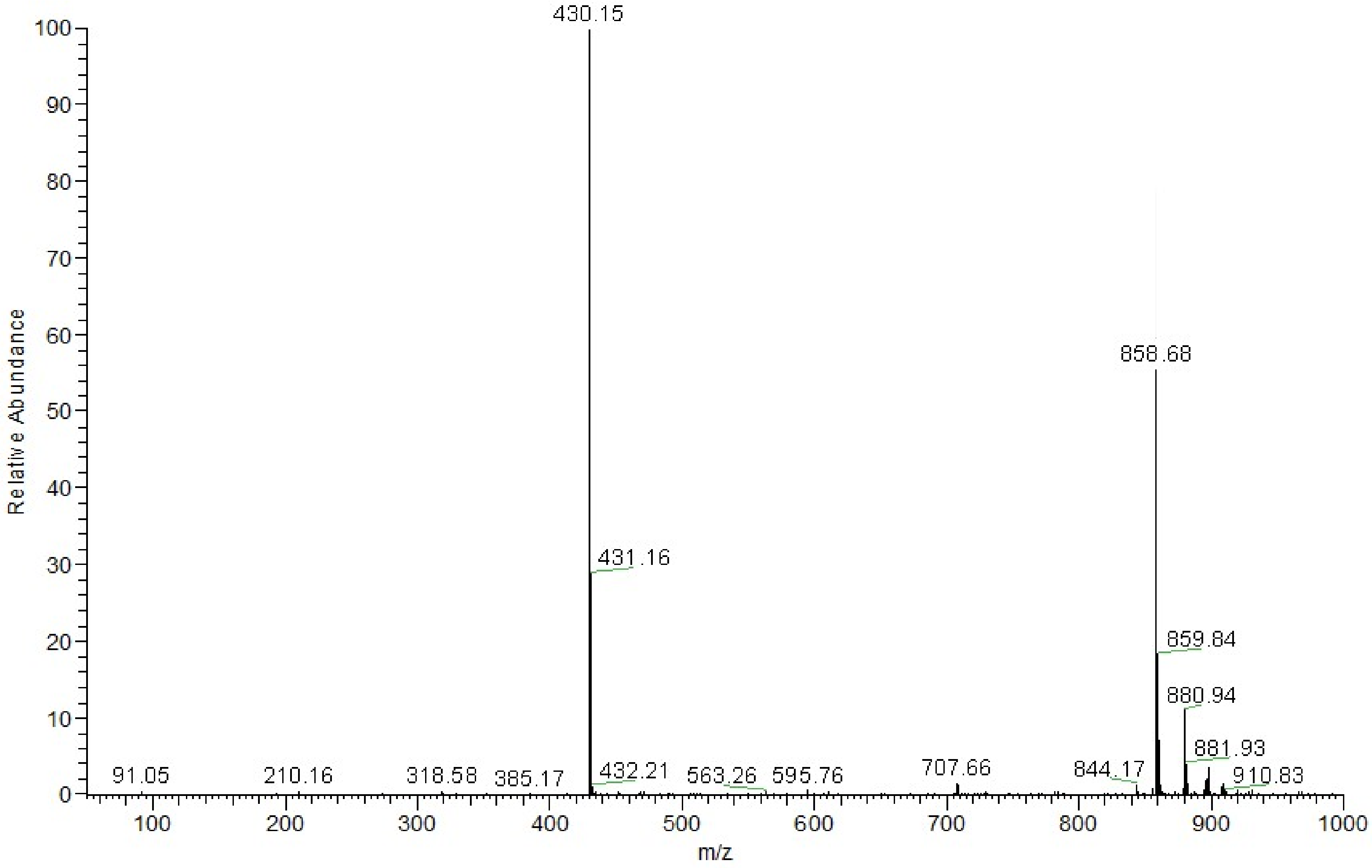

Figure S10. Mass spectra of compound **1b**.

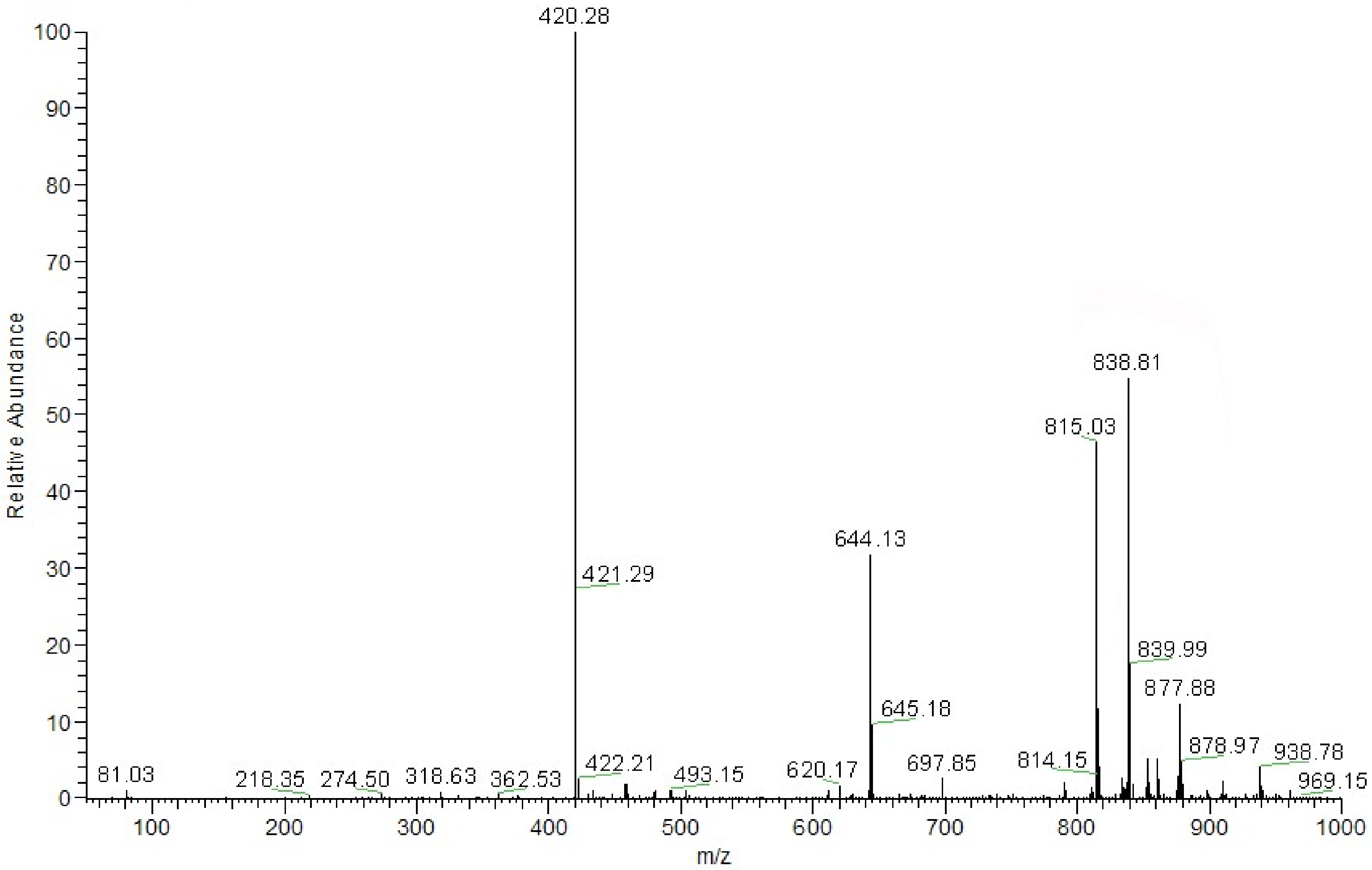

Figure S11. Mass spectra of compound **1c**.

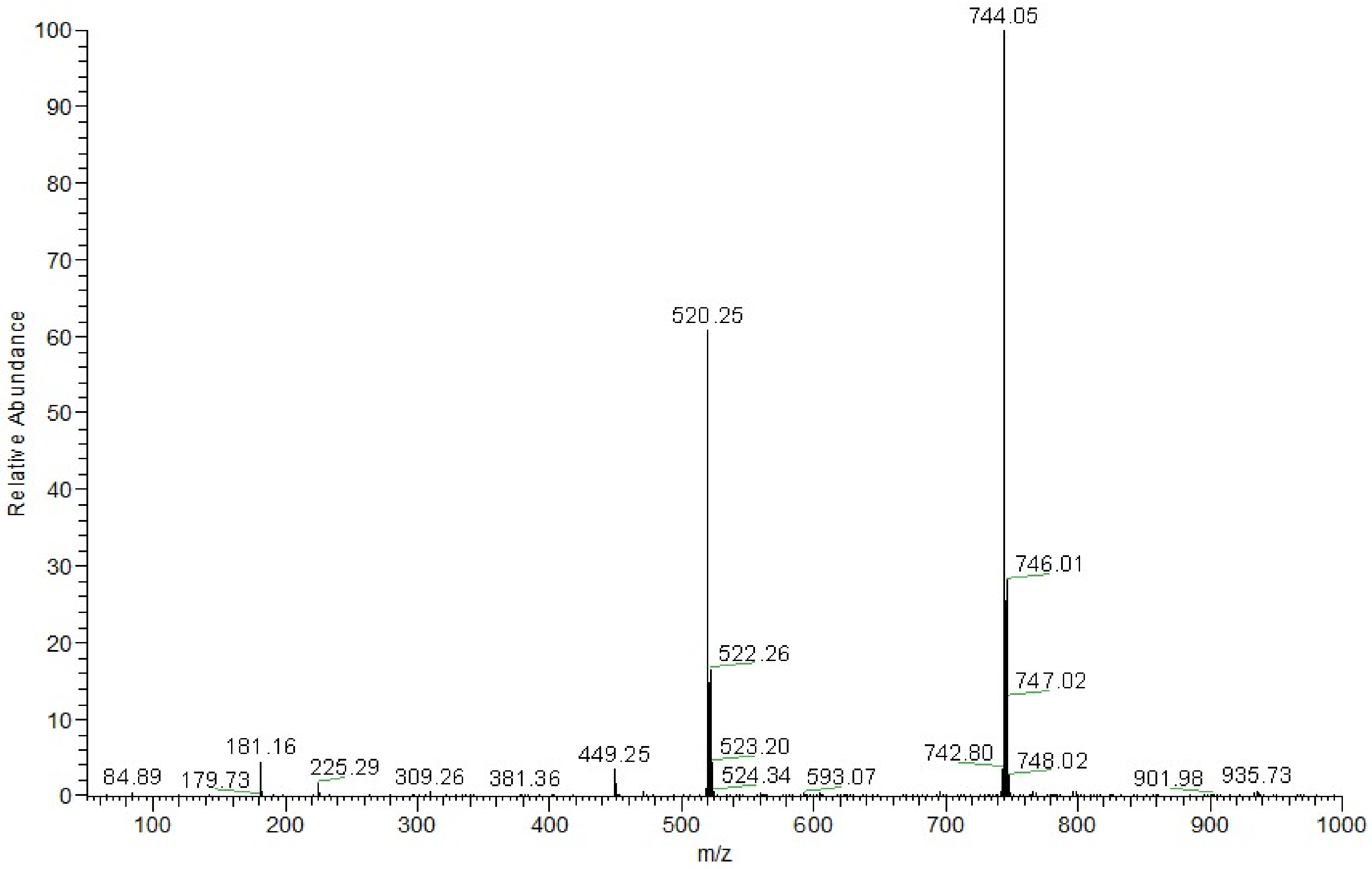

Figure S12. Mass spectra of compound **1d**.

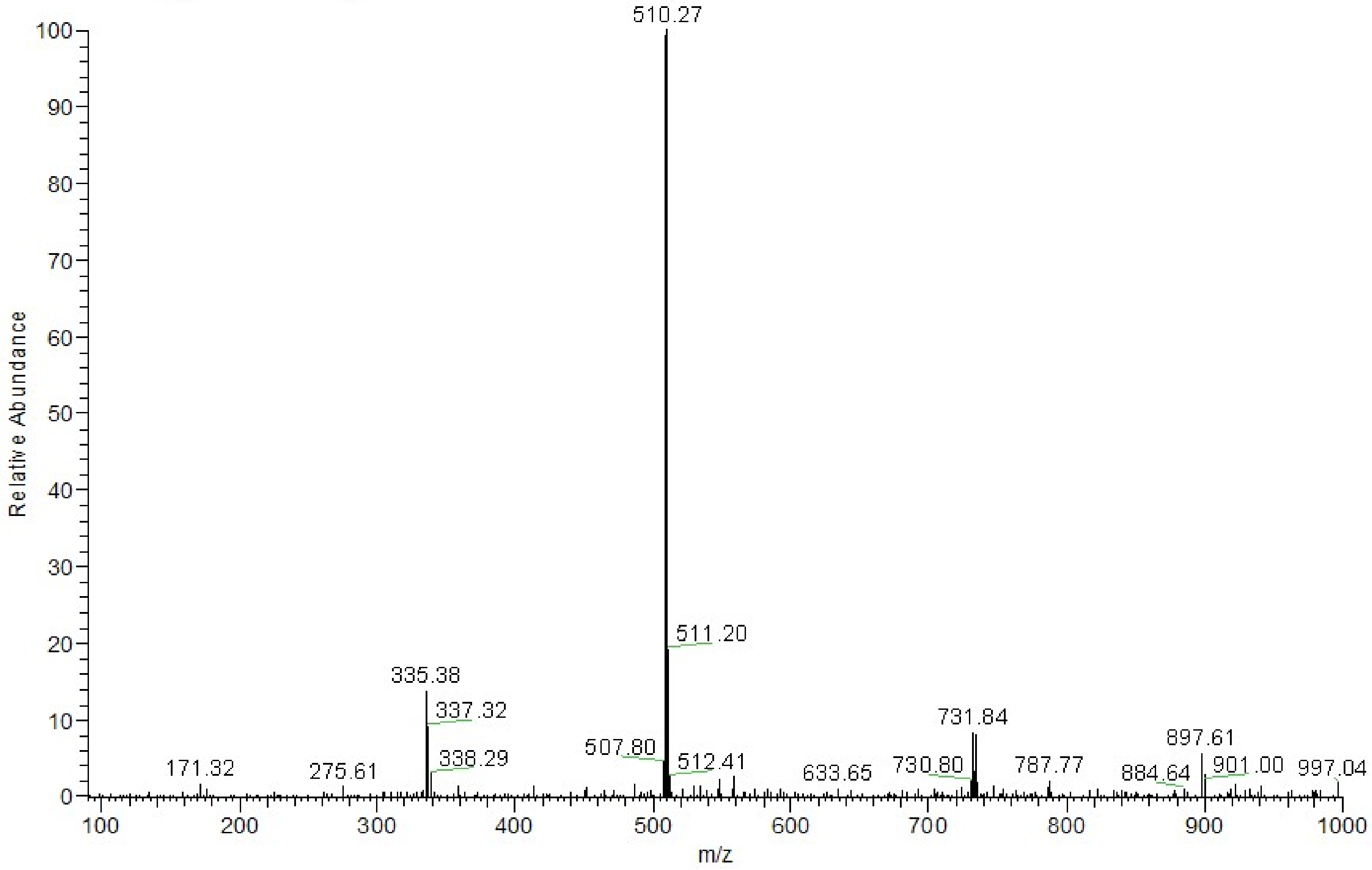

Figure S13. Mass spectra of compound **1e**.

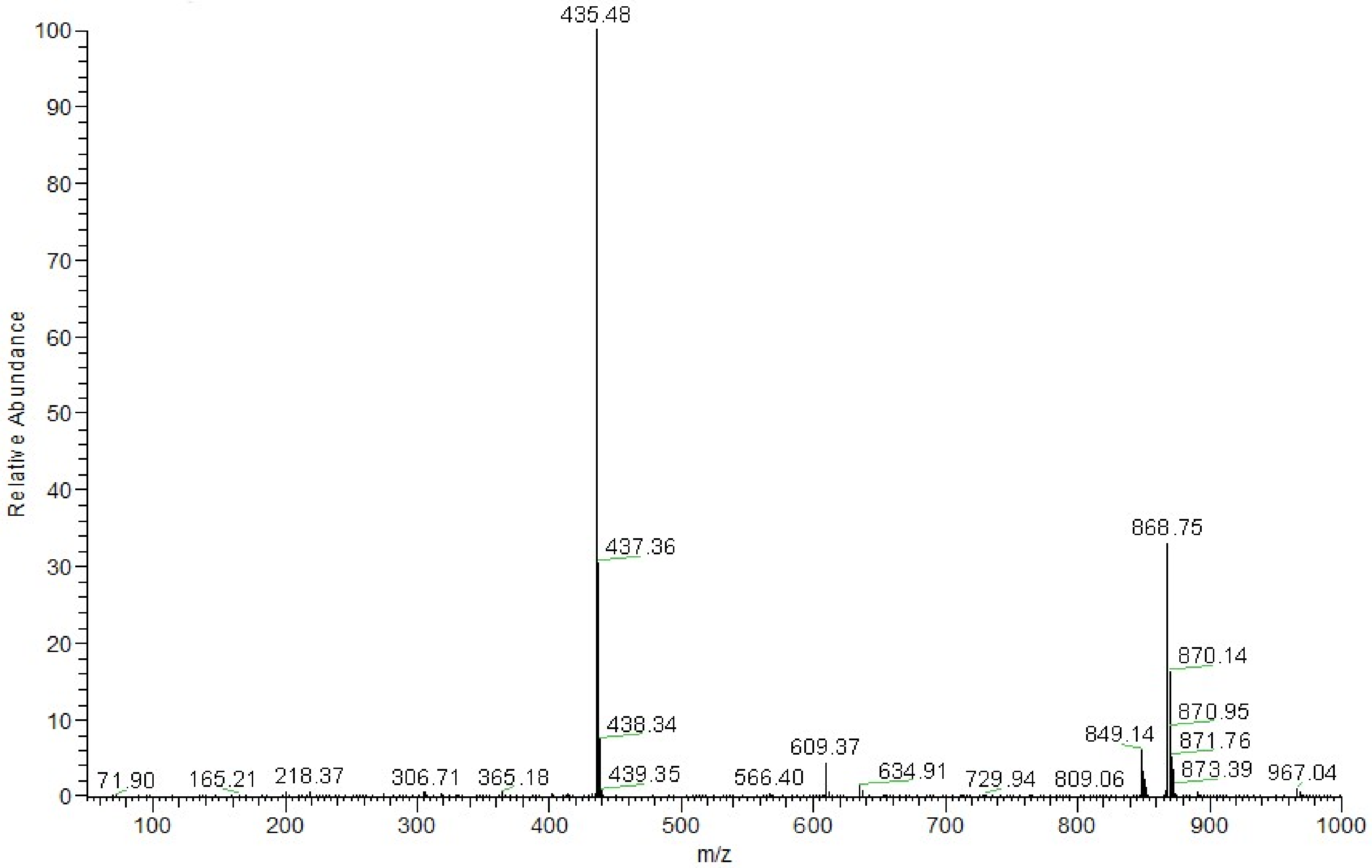

Figure S14. Mass spectra of compound **1f**.

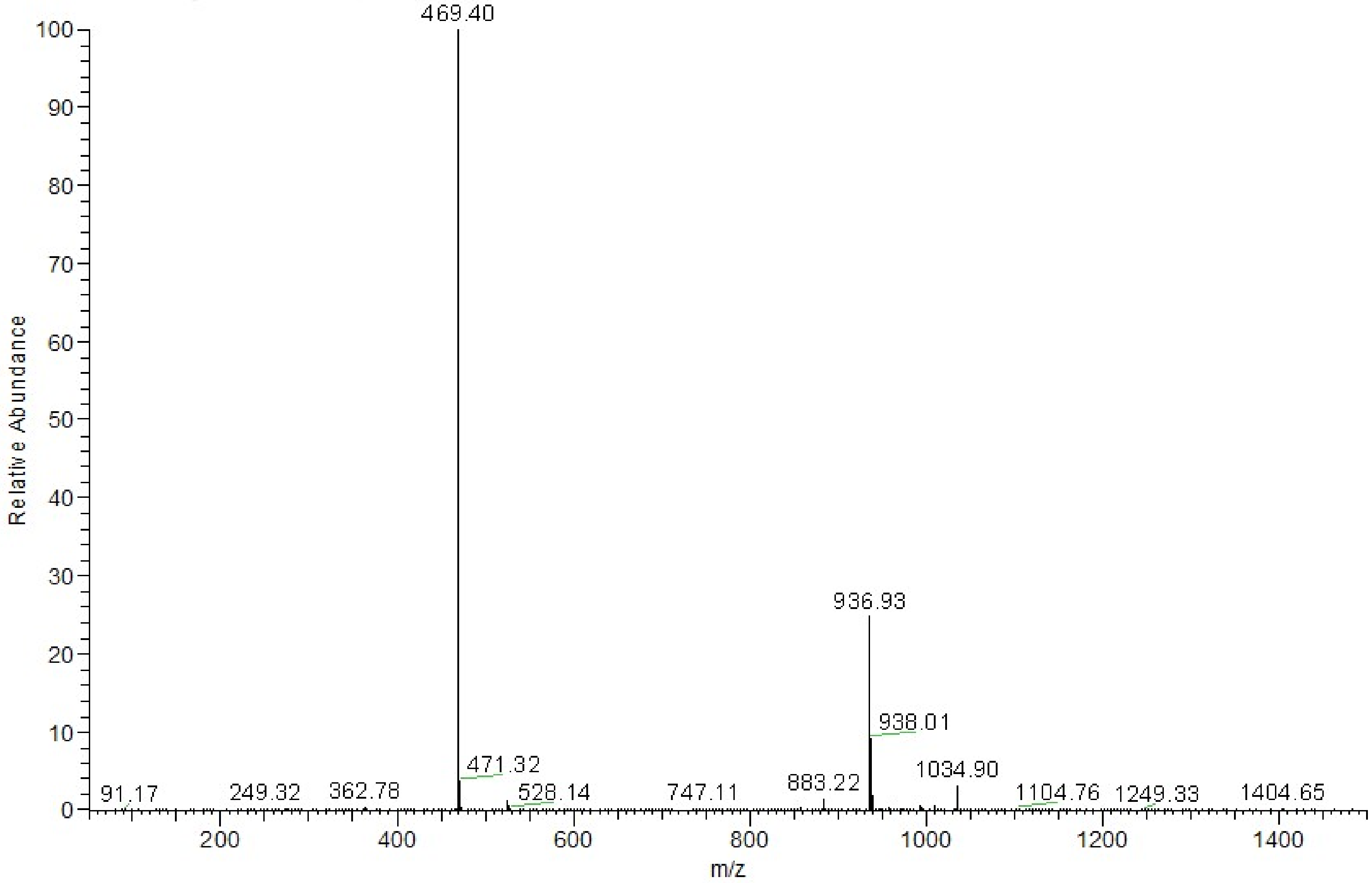

Figure S15. Mass spectra of compound **1g**.

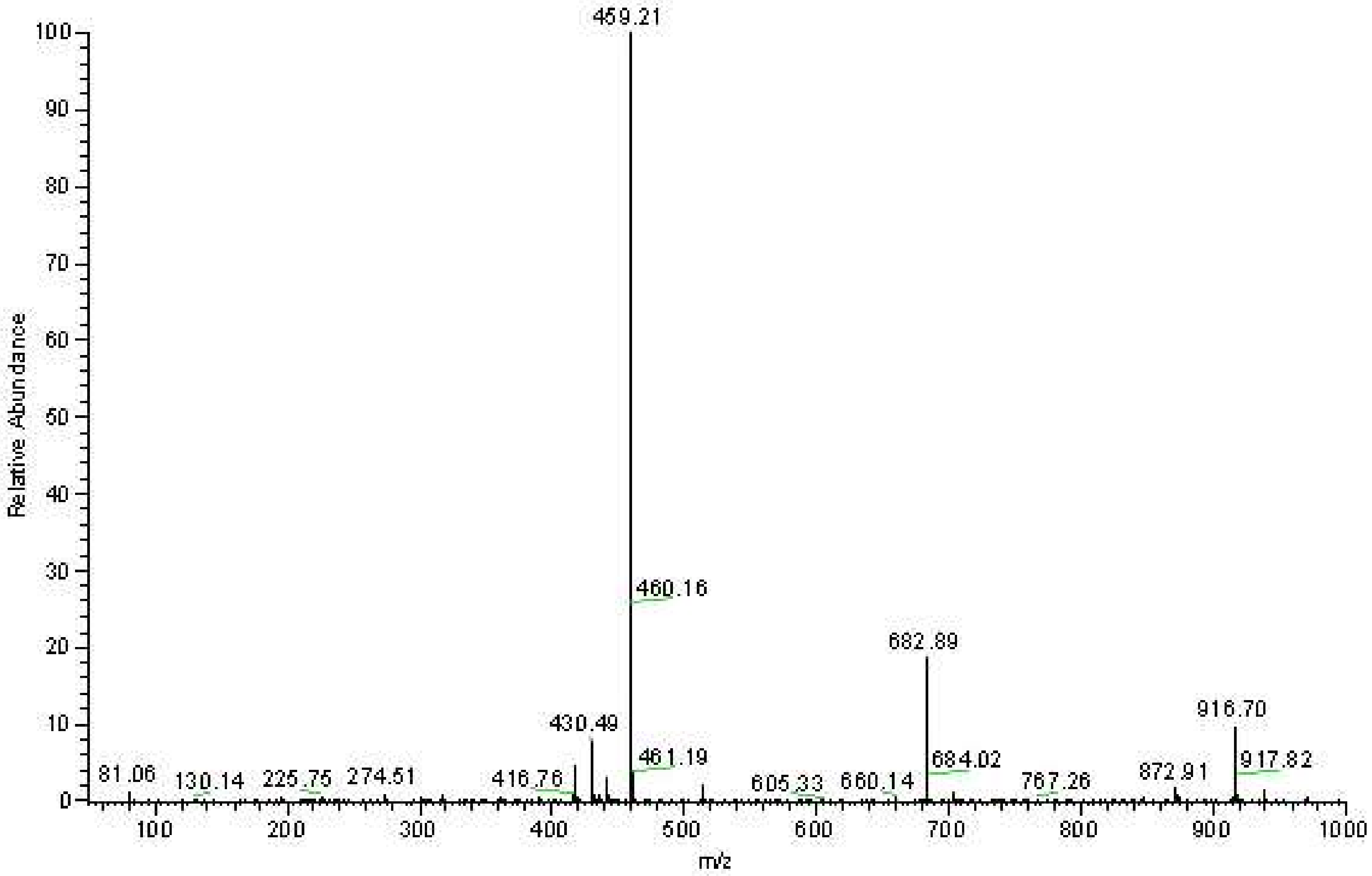

Figure S16. Mass spectra of compound **1h**.

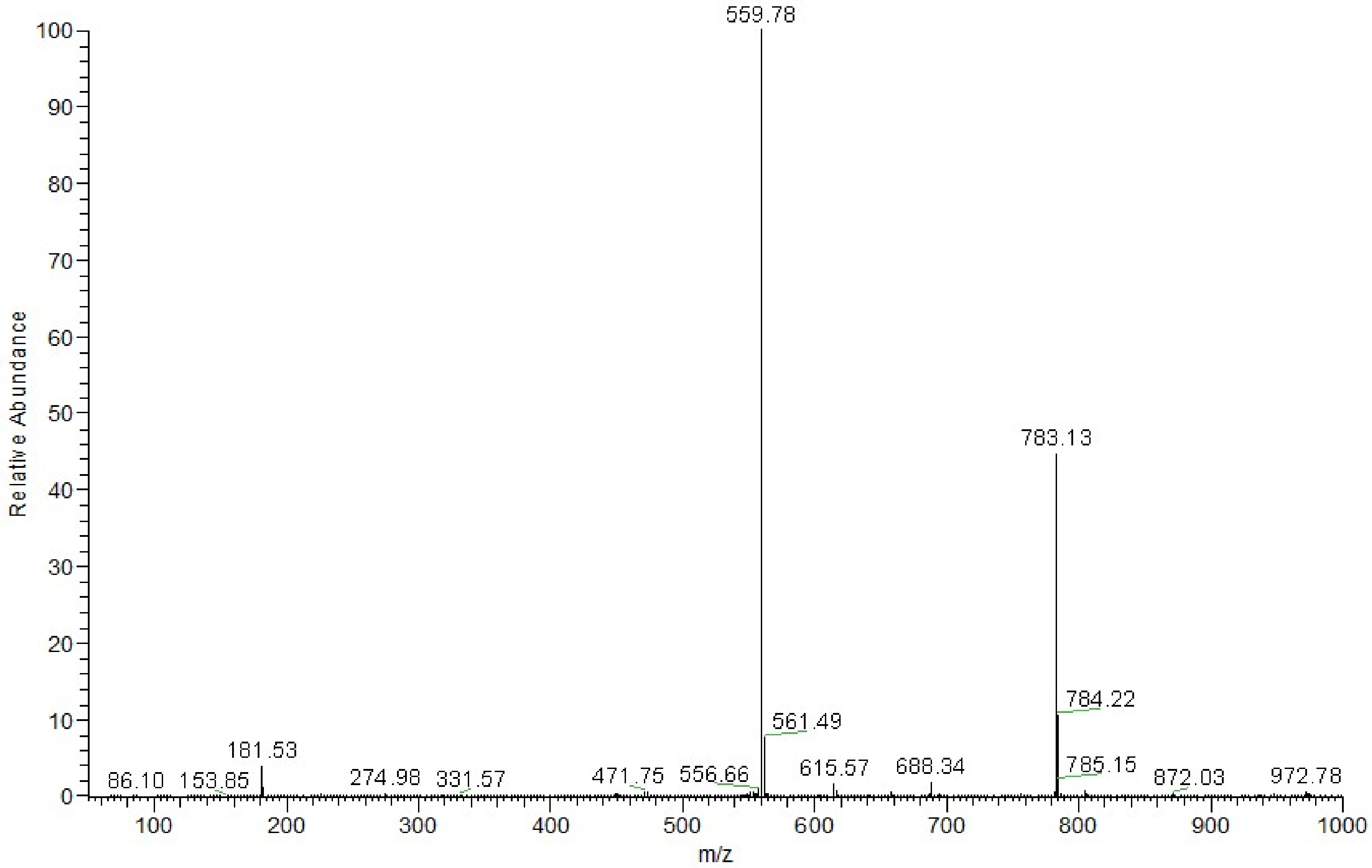

Figure S17. Mass spectra of compound **1i**.

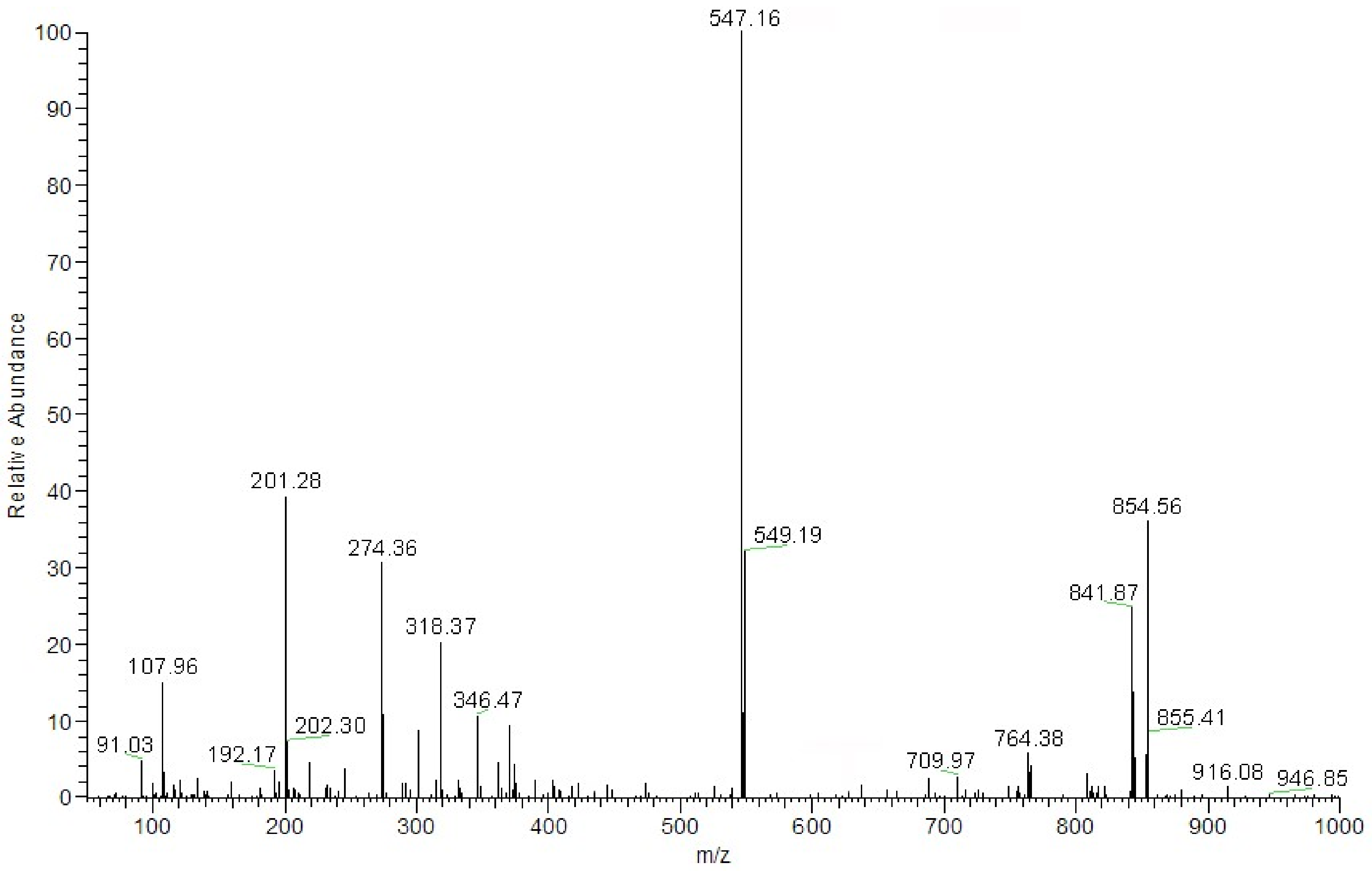

Figure S18. Mass spectra of compound **1j**.

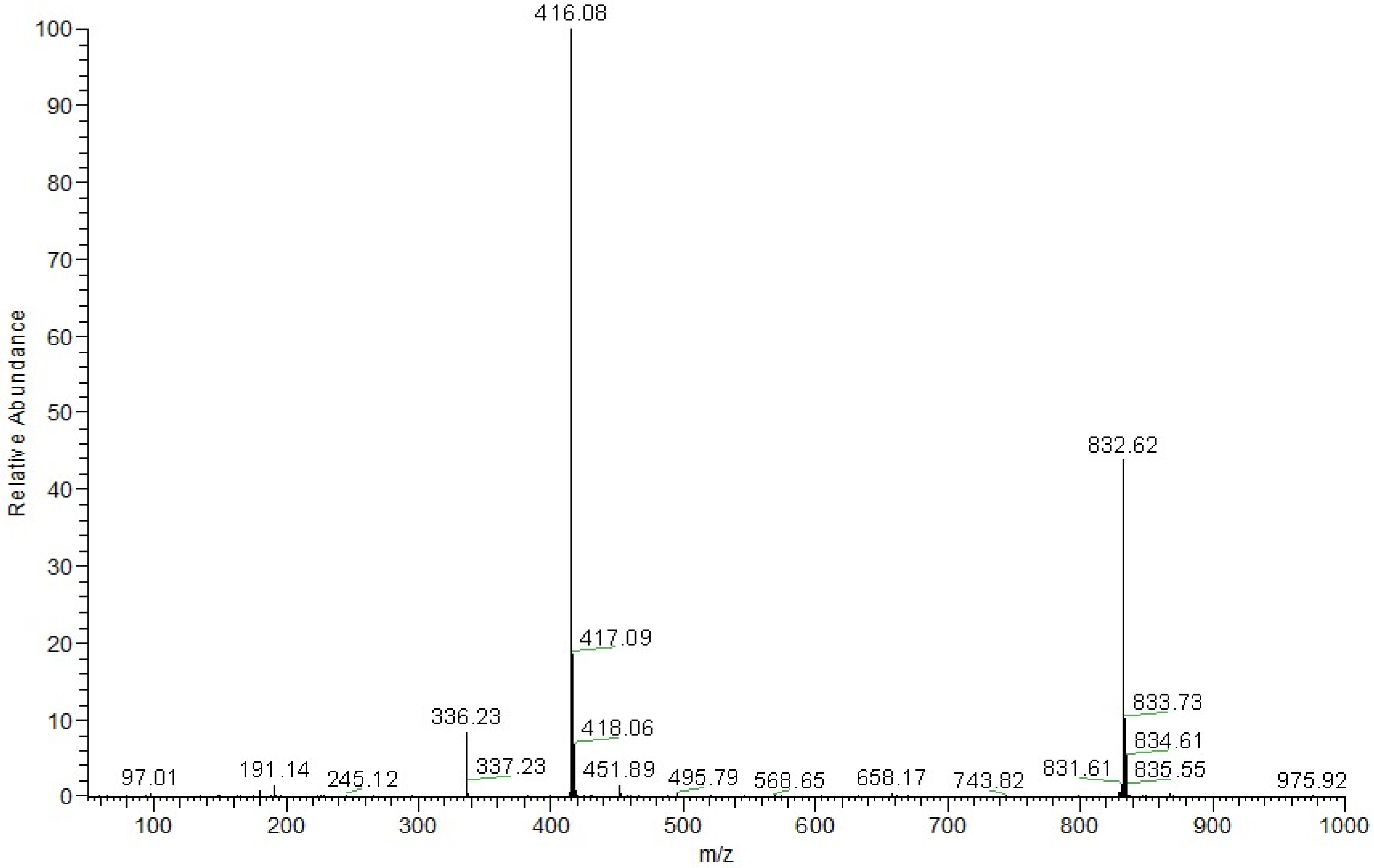

Figure S19. Mass spectra of compound **2a**.

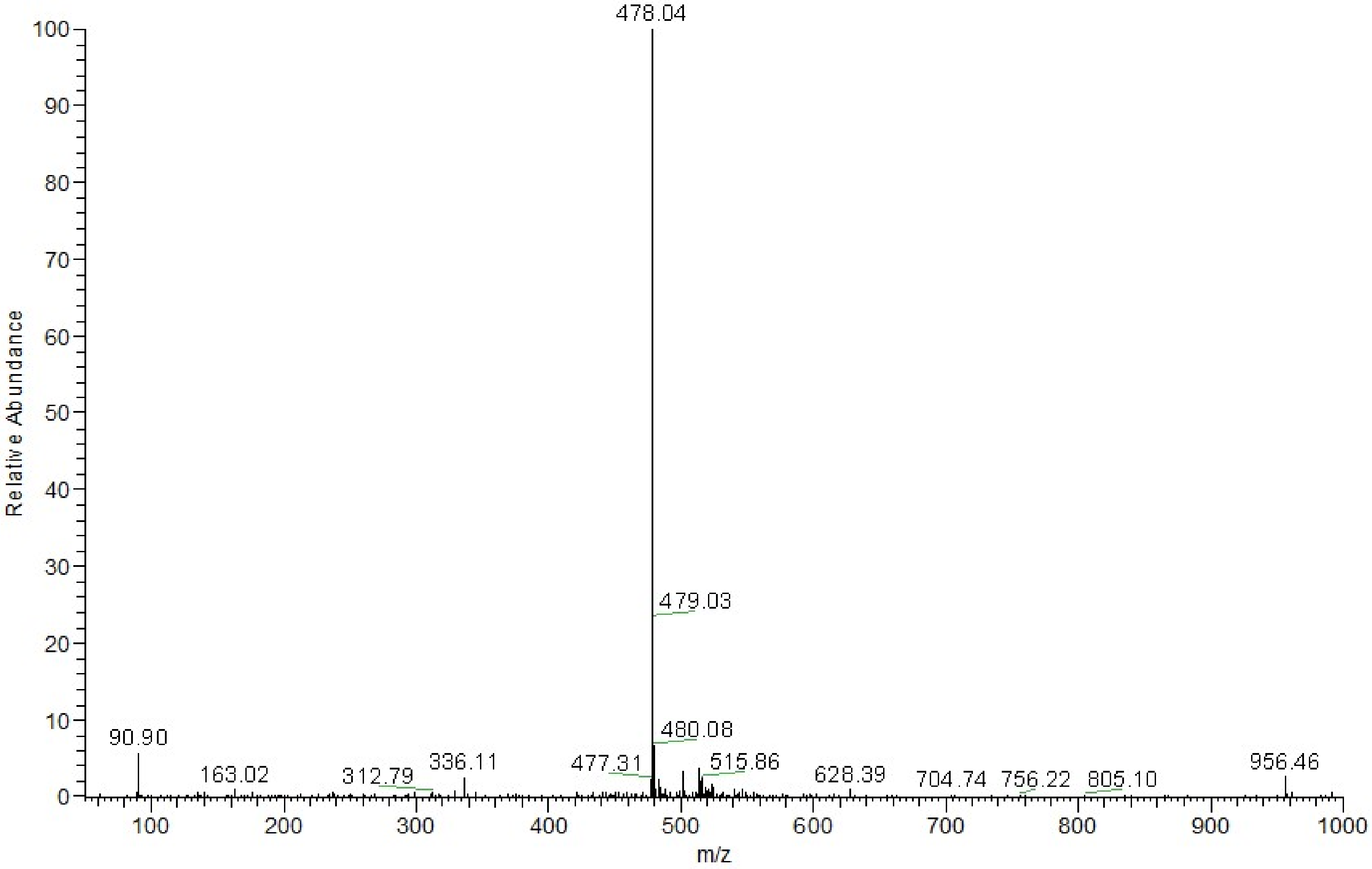

Figure S20. Mass spectra of compound **2b**.

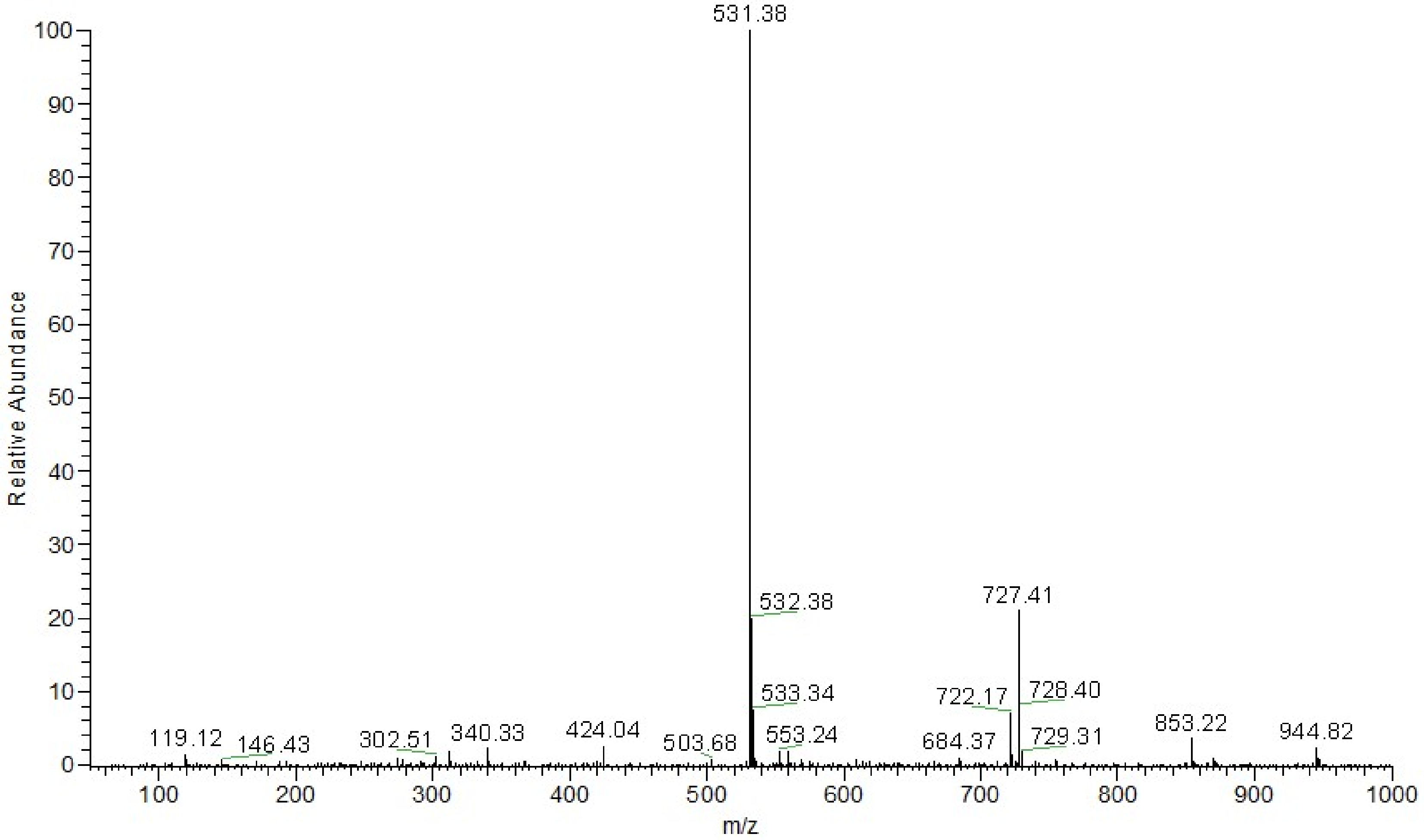

Figure S21. Mass spectra of compound **2c**.

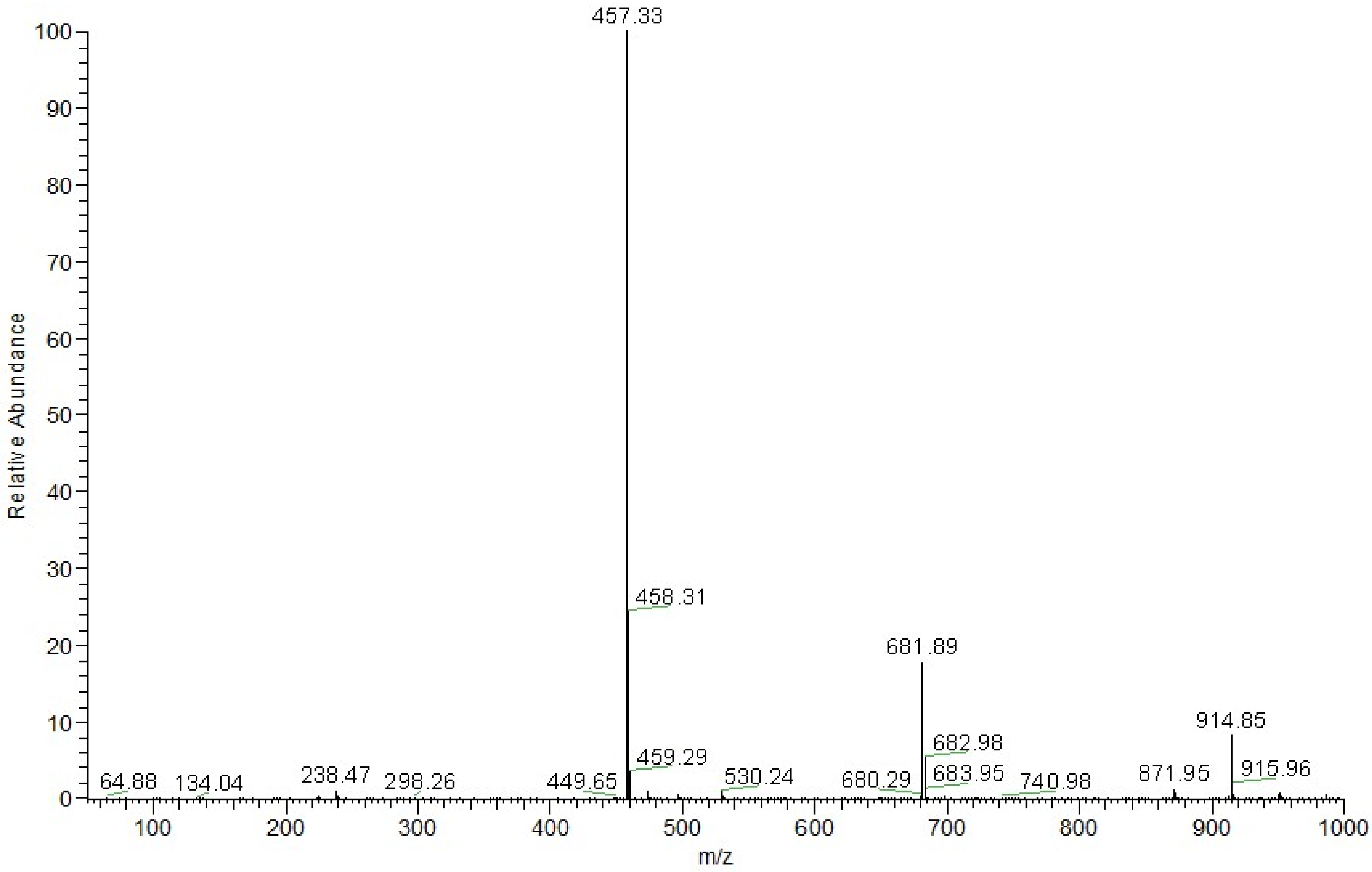

Figure S22. Mass spectra of compound **2d**.

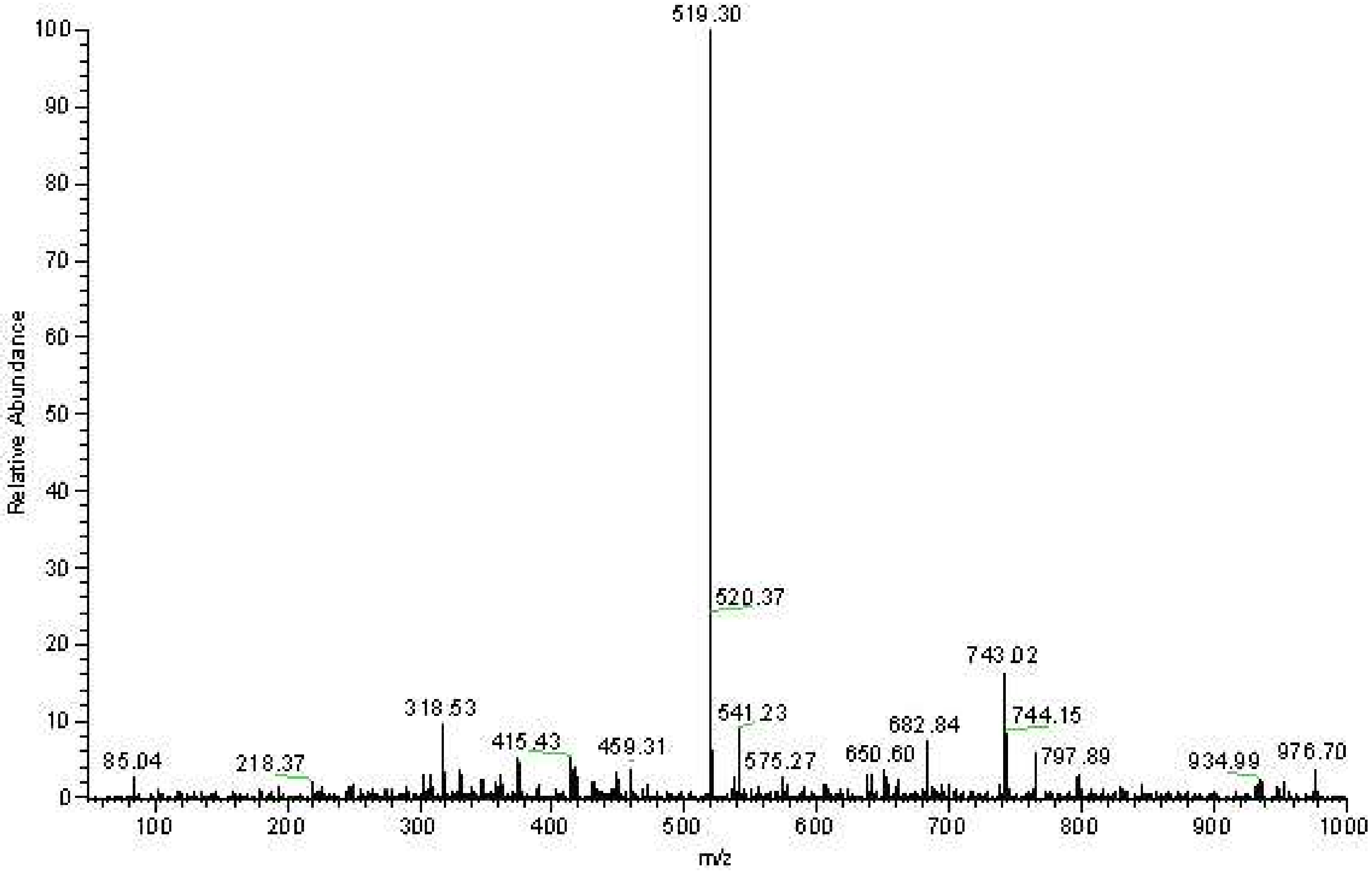

Figure S23. Mass spectra of compound **2e**.
